# Feasibility of the MAINTAIN intervention to support independence after a fall for people with dementia: a pilot cluster randomised controlled trial in participants’ own homes

**DOI:** 10.1136/bmjopen-2025-112336

**Published:** 2026-02-10

**Authors:** Leanne Greene, James Connors, Claire Hulme, Obioha C Ukoumunne, Robert Barber, Alison Bingham, Simon Conroy, Chris Fox, Carol Duff, Victoria Goodwin, Adam L Gordon, Abigail J Hall, Rowan H Harwood, Thomas Jackson, Rachael Litherland, Sarah Morgan-Trimmer, Steve W Parry, Ashima Sharma, Bethany Whale, Louise Allan

**Affiliations:** 1 University of Exeter Medical School, University of Exeter, Exeter, UK; 2 Exeter Clinical Trials Unit, University of Exeter, Exeter, UK; 3 NIHR ARC South West Peninsula, University of Exeter, Exeter, UK; 4 Campus for Ageing and Vitality, Cumbria Northumberland Tyne and Wear NHS Foundation Trust, Newcastle upon Tyne, UK; 5 Barts Health & Queen Mary University of London, London, UK; 6 Lincolnshire Partnership NHS Foundation Trust, Lincoln, UK; 7 Centre for Primary Care, Queen Mary University of London Wolfson Institute of Population Health, London, UK; 8 Academic Centre of Healthy Ageing, Barts Health NHS Trust, London, UK; 9 University of Nottingham School of Health Sciences, Nottingham, UK; 10 Health Care of Older People, Nottingham University Hospitals NHS Trust, Nottingham, UK; 11 Department of Inflammation and Ageing, School of Infection, Inflammation and Immunology, College of Medicine and Health, University of Birmingham, Birmingham, UK; 12 Department of Healthcare for Older People, Queen Elizabeth Hospital Birmingham, Birmingham, UK; 13 Innovations in Dementia, Exeter, UK; 14 School of Primary Care, University of Southampton, Southampton, UK; 15 Newcastle Upon Tyne Hospitals NHS Foundation Trust, Newcastle upon Tyne, UK

**Keywords:** Dementia, Aged, Aged, 80 and over, Aging, Frail Elderly, Rehabilitation medicine

## Abstract

**Objectives:**

To evaluate the feasibility of conducting a full-scale randomised controlled trial to assess the clinical and cost-effectiveness of the MAINTAIN intervention, designed to support recovery and independence following a fall among people living with dementia.

**Design:**

Pilot cluster randomised controlled trial (c-RCT).

**Setting:**

Community-based healthcare services across six UK sites representing primary and secondary care settings.

**Participants:**

31 participant-carer dyads were recruited. Eligibility criteria included a diagnosis of dementia and a recent fall. Exclusion criteria included severe comorbidity precluding participation. The consent rate was 84%, and retention at follow-up was 81%.

**Interventions:**

The MAINTAIN intervention comprised tailored, home-based therapy sessions delivered by trained professionals, focusing on functional recovery, confidence and re-engagement in daily activities, compared with usual care. The intervention was delivered over 12 weeks with booster sessions up to week 24, with the full trial period lasting 28 weeks.

**Primary and secondary outcome measures:**

Feasibility outcomes included recruitment and retention rates, intervention adherence and data completeness for outcome and economic measures. Exploratory outcomes assessed functional performance and quality of life. Feasibility outcomes were assessed at baseline, 12 weeks and 28 weeks.

**Results:**

Recruitment occurred over an 8-month period (September 2023–April 2024) across six UK sites. Most intervention participants (89%) attended at least 60% of planned sessions. Completion rates for outcome and economic data were high, indicating strong acceptability and feasibility of both the intervention and trial procedures.

**Conclusions:**

The pilot c-RCT demonstrated that recruitment, retention and intervention delivery were feasible and well accepted. Findings support progression to a definitive trial to evaluate the effectiveness and cost-effectiveness of the MAINTAIN intervention.

**Trial registration number:**

ISRCTN16413728 (International Standard Randomised Controlled Trial Number registry).

**Strengths and limitations of this study**

- A cluster randomised controlled design was used to test trial procedures and intervention delivery in real-world dementia care settings.
- Multiple health service sites were included, allowing assessment of recruitment pathways and infrastructure needs across different contexts.
- Economic and quality-of-life data collection methods were evaluated for feasibility, including self-completed and proxy-completed EuroQol 5-Dimension 5-Level Instrument and service-use questionnaires.
- Cluster randomisation before participant enrolment introduced potential for baseline imbalance and selection bias, suggesting an individually randomised design may be preferable in a definitive trial.
- Falls data collection methods (eg, diaries) were limited by cognitive and practical challenges, highlighting the need for more pragmatic approaches in future trials.

## Introduction

 People living with dementia have a higher risk of falls than those without,^1 2^ and post-fall outcomes are often worse, with poorer recovery trajectories.^3^ Consequences extend beyond injury to reduced independence,^4^ loss of confidence and fear of future falls.^5 6^

In the UK, ~9 82 000 people live with dementia,^7^ around 60% at home.^8^ Those at home may have less access to formal fall-prevention services and greater reliance on informal care, heightening risk after a fall. Falls cost the UK health and social care ~£4.4 billion annually.^9^ Despite rising prevalence and elevated fall risk,^1 2^ evidence for dementia-specific fall interventions in community-dwelling populations remains limited.^10^

Trials of fall-related interventions in dementia show mixed results: some improve physical function, but clinical outcomes remain inconsistent.^11^,^14^ National Institute for Health and Care Excellence (NICE guidelines also lack dementia-specific fall-prevention recommendations.^15 16^ To address this, the Developing an Intervention for Fall-Related Injuries in Dementia (DIFRID) programme was created and evaluated.^17^ MAINTAIN was developed as a refined version of the DIFRID programme. As a home-based, multicomponent rehabilitation programme, it represents a complex intervention; consistent with the updated Medical Research Council (MRC) guidance,^18^ its design reflects the need to address the multiple interacting factors that influence recovery after a fall in dementia, including physical capability, confidence, daily routines and caregiver involvement. Its development was informed by components shown to support these factors, such as frequent home visits, falls education, tailored functional activity, caregiver-supported practice and multidisciplinary input, all of which are expected to enhance independence after a fall. This draws on evidence that routine-building, person-centred goal-setting and embedding strength and balance activity into everyday tasks can improve engagement, confidence and functional ability in people with dementia.^19^,^21^ See Greene *et al* for more detail on the development of the intervention/programme theory.^22^ The objectives of this pilot were to evaluate MAINTAIN’s feasibility through a cluster randomised controlled trial (c-RCT) with process evaluation, develop a cost-effectiveness framework, refine the intervention for a full trial and assess risks to allocation concealment, including potential unblinding and recruitment or retention imbalances between trial arms.

## Methods

### Patient and public involvement

Our patient and public involvement and engagement (PPIE) panel comprised people living with dementia and carers. The panel was involved from the earliest stages of the research. Individuals with lived experience helped shape the research question and contributed to the development and refinement of the MAINTAIN intervention. Their priorities directly informed the focus of the study; in particular, they emphasised the importance of supporting people to maintain independence following a fall, an area they identified as under-researched.

Panel members were recruited through the Community Interest Company *Innovations in Dementia*. They contributed to the design of participant information sheets, consent forms and other study materials, monitored recruitment and advised on recruitment strategies. They also reviewed the analysis of the process evaluation, which is reported separately.^22^

### Study design

Participating sites were chosen to ensure a diverse range of potential participants in terms of ethnicity, socioeconomic status and rural or urban location. The UK National Health Service (NHS) and local authority research governance and research ethics committee approved all study procedures (NHS REC reference: 23/WA/0126), and the trial was performed in accordance with the ethical standards as laid down in the 1964 Declaration of Helsinki and its later amendments or comparable ethical standards. Written informed consent was obtained from participants. The study protocol^23^ and embedded process evaluation^22^ have been published. The reporting of findings followed the Consolidated Standards of Reporting Trials (CONSORT) for pilot or feasibility trials^24^ (online supplemental file 1). Trial registration number: ISRCTN16413728. Study design, conduct and reporting plans were informed by regular consultation with the PPIE panel and key stakeholders.

### Randomisation and blinding

A cluster randomised design was chosen to minimise potential contamination, as therapists delivering MAINTAIN may also have had routine clinical responsibilities within their services, creating a risk that intervention approaches could inadvertently influence the care of control participants.

Sites (clusters) were randomised before recruitment to allow intervention services time to prepare. The allocation sequence was generated by the trial statistician using a random seed, entered into the Research Electronic Data Capture (RedCap) and applied within RedCap to ensure concealment before randomisation.

Data were collected at baseline, 12 weeks and 28 weeks. As this was a cluster randomised trial, research staff necessarily knew site allocation. Owing to the nature of the intervention, therapists and participants could not be blinded, and clinical researchers collecting follow-up data were also unblinded, particularly when recording 12-week health-utilisation data that indicated treatment allocation. To minimise recruitment bias, participants were blinded to allocation until consent. Statisticians remained blinded until finalisation of the Statistical Analysis Plan (SAP) to reduce the risk of analytical bias. The SAP is provided in online supplemental file 2.

### Referral and recruitment methods

Recruitment ran from 1 September 2023 to 30 April 2024 across six healthcare and research services, reflecting the varied structures of dementia care in the UK (online supplemental file 3). Several sites were embedded within NHS Trusts providing mental health, community or rehabilitation services, supported by the National Institute for Health and Care Research Delivery Teams and Clinical Research Network staff. Some used dedicated research registers, while others identified participants via general practitioner (GP) surgeries, memory and falls clinics or rapid access services.

One additional site was a charitably funded service for people with dementia and carers, which recruited through prior service users and clinician referrals. Participants were identified and recruited by healthcare staff employed within the participating NHS Trusts, including research nurses, assistant psychologists, mental health nurses and research practitioners. These staff were not involved in participants’ clinical care or in delivering the intervention. Further individual site detail is provided in online supplemental file 5.

### Study population and participant eligibility criteria

Participants were aged ≥50 years, diagnosed with dementia and listed on the Primary Care Quality Outcomes Framework register. The research team confirmed diagnoses within 4 weeks of identification. Inclusion required at least one fall in the previous 6 months, defined as an event where the person came to rest on the ground or a lower level, with or without loss of consciousness.

Eligible participants lived in their own homes at the time of the index fall and intervention; those in care homes were excluded. Each participant needed an unpaid carer willing to participate. Individuals with dementia either provided consent or, if lacking capacity, a personal/nominated consultee did so under the Mental Capacity Act (2005).

Carers were identified by the person with dementia and their family or friends. They were required to have contact for ≥1 hour per week, the capacity to consent and to communicate in English.

### Intervention procedures

MAINTAIN was a personalised, home-based, multidisciplinary programme addressing the physical and psychosocial needs of people with dementia. The initial session focused on collaborative goal setting with therapists, participants and unpaid carers. Up to 19 sessions were delivered over 12 weeks, plus three booster sessions at weeks 16, 20 and 24 (maximum 22 sessions over 28 weeks; online supplemental file 4). Delivery followed a manual and therapist training.

Therapists completed online or face-to-face training led by physiotherapists and occupational therapists with dementia expertise. Content included dementia awareness, communication, person-centred care, risk reduction, pain management and Specific, Measurable, Achievable, Relevant, and Time-bound (SMART) goal setting using Goal Attainment Scaling.^25^

### Assessment sessions

The first session used a structured proforma to record falls history, comorbidities, medications, living arrangements, mobility, activity, home safety and risk factors such as fear of falling; carer stress was also assessed. Physical and functional tests, including the Timed Up and Go (TUG)^26^ and a home environment review, informed a personalised problem list and goals. A multidisciplinary team then created an action plan, arranged referrals (eg, GPs, community groups) and addressed carer support needs.

### Therapy sessions

Sessions lasted up to 60 min and were tailored to progress, with up to three delivered by a physiotherapist, three by an occupational therapist (OT) and the remainder by a rehabilitation support worker (RSW). Activities included strength and balance training, dual-task exercises and functional tasks integrated into daily routines (eg, balancing while washing up). Unpaid carers supported goal setting and activity promotion, with cueing cards and illustrations used to aid adherence.

Each session was documented using a structured proforma to record activities, recommendations and adherence. Non-adherence was reviewed and goals adjusted. After the final session, a summary and recommendations were sent to the participant’s GP.

### Outcomes

The primary objective of this pilot c-RCT was to assess the feasibility of conducting a definitive trial of the MAINTAIN intervention for community-dwelling people with dementia who had experienced a fall. Feasibility outcomes included recruitment and retention rates, intervention adherence, data completeness and the acceptability and delivery of trial procedures.

The proposed primary outcome for a future definitive trial is functional independence, assessed using the Disability Assessment for Dementia (DAD). Secondary objectives of the pilot were to test the cost-effectiveness framework, refine the intervention and identify threats to allocation concealment, including potential preconsent unblinding, recruitment disparities, baseline imbalances and differential follow-up.

To support these objectives, a range of participant-level measures were collected. Secondary outcome measures included quality of life (EuroQol 5-Dimension 5-Level Instrument (EQ-5D-5L); Quality of Life-Alzheimer’s Disease and carer-rated proxies),^27^ Short Falls Efficacy Scale – International,^28^ mobility (TUG)^26^ and carer burden (Zarit Caregiver Burden Assessment: Zarit Burden Interview-12).^29^ Goal Attainment Scaling was completed in the intervention arm.^25^ Falls were recorded using carer-supported diaries, and health and social care utilisation was captured via a questionnaire.

### Sample size

Over 6 months, each of six sites was expected to recruit 10 dyads (person with dementia plus carer), giving 60 dyads (30 intervention, 30 control). Based on prior work,^17^ the anticipated recruitment rate was 1.7 dyads per site per month.

The sample size was chosen to provide precise estimates of feasibility. Of 150 eligible individuals, a 40% consent rate was expected, yielding 60 participants; this gives a 95% CI for consent of 29% to 51%. Assuming 80% follow-up, retention would be 66% to 91% (95% CI). In the intervention arm, 30 participants were sufficient to estimate ≥60% session attendance, assumed at 80%, with a 95% CI of 60% to 93%. Calculations accounted for clustering, using an intracluster correlation coefficient of 0.05.

### Statistical analysis

Baseline characteristics of services and participants were summarised as means (SD) for continuous variables and frequencies (%) for categorical variables. With 95% CIs accounting for clustering, we reported: percentage eligible, percentage consenting, percentage providing follow-up data and percentage of intervention participants attending ≥60% of sessions.

Binary outcomes (consent, follow-up, attendance) were analysed using mixed effects logistic regression with Satterthwaite’s correction. Model-derived constants (log odds) were converted to percentages with 95% CIs. SD estimates for continuous outcomes were calculated at baseline, 12 weeks and 28 weeks. To assess recruitment bias from cluster randomisation and unblinding, eligibility and participation rates were compared between arms, alongside baseline characteristics.

Secondary analyses estimated intervention effects on continuous outcomes at 12 weeks and 28 weeks, adjusted for baseline scores. Mixed effects linear regression with Satterthwaite’s correction was used to account for the small number of clusters. Analyses were conducted in Stata.

### Economic evaluation

The study assessed the acceptability and completeness of resource-use and outcome data to inform the design of a future cost-effectiveness analysis. The evaluation adopted a societal perspective, considering direct and indirect effects,^30^ and included outcomes for both people with dementia and carers, consistent with NICE guidance recommending inclusion of patient and caregiver effects.^15^

Cost-effectiveness used the EQ-5D-5L.^31^ Participants with capacity completed self-reports; carers provided proxy responses plus their own EQ-5D-5L. Index scores were generated using the English value set recommended by NICE. The proportion of missing or incomplete responses was recorded for both self- and proxy-reported questionnaires.

A resource-use questionnaire, adapted from prior dementia studies^32 33^ and refined with PPIE input (March 2023), captured healthcare, social care, informal support and out-of-pocket costs. It was administered at baseline, 12 weeks and 28 weeks, with completion rates and data quality summarised descriptively.

### Success criteria and barriers to success

Success criteria and barriers to success are outlined in online supplemental file 5.

## Results

### Recruitment, retention and dropout rate

62 older adults were screened; 25 were excluded. Common reasons were participant/consultee declining (7/52), no suitable unpaid carer (6/58), carer declining (5/56) or no fall in the past 6 months (4/61). Other reasons included no dementia diagnosis on the Quality and Outcomes Framework register (1/55), not living at home during fall and intervention (1/58) and lacking capacity without a consultee (3/52).

Eight potential participants declined (lack of perceived benefit, n=3; time burden, n=2; no interest, n=1; other, n=2). Two consultee referrals were not enrolled as consultees advised non-participation. Five carers declined (lack of perceived benefit, n=2; time burden, n=1; other, n=2). Declines occurred both pre-eligibility and post-eligibility confirmation.

Of 37 eligible, 31 consented and were randomised by cluster (intervention n=18; control n=13). 25 dyads completed the trial. Eight withdrew: six full dyads and two participants only (carers continued data). Reasons were personal (n=4), lack of benefit (n=1), no available carer (n=1) and other (n=2).

The trial ran at six sites, four of which recruited successfully. All randomised participants received their allocated intervention.

### Baseline characteristics

Table 1 shows participant characteristics. In the intervention arm (n=18), all were white; mean age was 77.8 years (SD 10.0; 60–93), with nine females and nine males. In the control arm (n=13), 11 were white, one Hispanic and one missing; mean age 79.2 years (SD 7.9; 62–90), with nine men and four women. Mean clinical frailty scores were 5.2 (intervention) and 4 (control).

**Table 1:** Patient demographics

Patient demographics	Control group		Maintain group	
	N	n (%)	N	n (%)
Dementia subtype	12		18	
Alzheimer’s disease		5 (41.7%)		7 (38.9%)
Vascular dementia		2 (16.7%)		2 (11.1%)
Mixed dementia		3 (25%)		8 (44.4%)
Dementia with Lewy bodies		2 (16.7%)		1 (5.6%)
Ethnicity	12		18	
White		11 (91.7%)		18 (100%)
Mixed/multiple ethnic groups		0 (0%)		0 (0%)
Asian/Asian British		0 (0%)		0 (0%)
Black/African/Caribbean/black British		0 (0%)		0 (0%)
Other		1 (8.3%)		0 (0%)
Sex	13		18	
Male		9 (69.2%)		9 (50%)
Female		4 (30.8%)		9 (50%)
Residence type	12		18	
Private home		10 (83.3%)		18 (100%)
Sheltered accommodation		1 (8.3%)		0 (0%)
Other		1 (8.3%)		0 (0%)
Living arrangement	12		18	
Lives alone		2 (16.7%)		2 (11.1%)
Lives with spouse/partner		10 (83.3%)		15 (83.3%)
Lives with other family member		0 (0%)		1 (5.6%)
Lives with someone other than family		0 (0%)		0 (0%)
Other		0 (0%)		0 (0%)
Does the participant speak/read and understand English?	12		18	
Yes		12 (100%)		18 (100%)
No		0 (0%)		0 (0%)
Highest education level	12		18	
Secondary school or equivalent		4 (33.3%)		9 (50%)
Sixth form/college/apprenticeship		7 (58.3%)		7 (38.9%)
University undergraduate		0 (0%)		1 (5.6%)
University postgraduate		1 (8.3%)		1 (5.6%)
Smoking status	12		18	
Never smoked		6 (50%)		8 (44.4%)
Ex-smoker		6 (50%)		9 (50%)
Current smoker		0 (0%)		1 (5.6%)

Carers in the intervention arm (n=18) had a mean age of 70.8 years (47–88), 10 female and eight male; 14 were spouses/partners and four sons/daughters. In the control arm (n=13), 11 were white, one Arab and one missing; mean age 70.7 (45–88) for 12 recorded, eight female and four male, with 10 spouses/partners and two sons/daughters. Ethnicity was self-reported.

### Feasibility and outcome results

#### Feasibility

**Figure 1:**
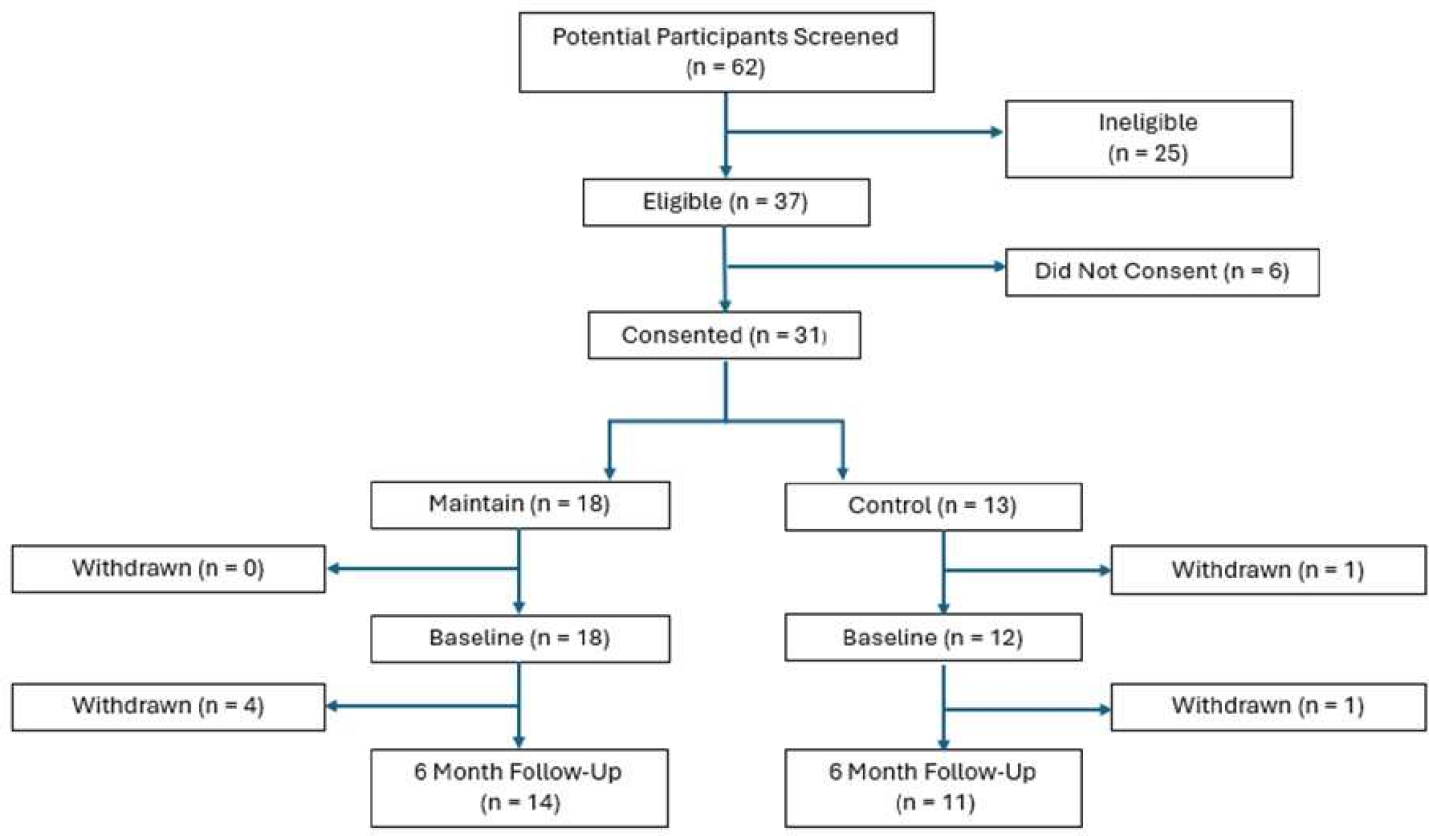
Consolidated Standards of Reporting Trials participant flow diagram.

62 potential participants were invited to take part in the MAINTAIN study, with 37 being identified as meeting the full eligibility criteria for participation. 31 dyads agreed to participate in the study (consent rate 84% (95% CI 68% to 94%)). The recruited sample was below our target number of 60 dyads. Two of the six study sites did not recruit any participants. 25 of the 31 participants completed the final follow-up (81% (95% CI 63% to 93%)). Intervention adherence was defined as attendance of 60% of the planned sessions; this was achieved by 16 (89% (95% CI 65% to 99%)) of the 18 participants in the intervention arm. See figure 1 for the CONSORT diagram.

#### Outcomes

At baseline, 30 participants completed the DAD: all 18 in the intervention arm (mean 54.3, SD 27.8) and 12 in the control arm (mean 71.1, SD 16.9). At 6 months, 25 participants completed the DAD: 14 in the intervention arm (mean 58.7, SD 32.9) and 11 in the control arm (mean 60.0, SD 23.8). Baseline differences reflected chance variation from small sample sizes.

Falls data were insufficient for analysis due to low diary returns, an influential outlier and no baseline measure. Mixed effects Poisson regression could not be fitted; descriptive statistics only are reported (tables2^4^).

**Table 2:** Outcome data

	Maintain group			Control group			Model statistics	
	N	Mean	SD	N	Mean	SD	Mean difference	95% CI
Activities of daily living assessed with the DAD at baseline	18	54.3	27.8	12	71.1	16.9		
Activities of daily living assessed with the DAD at 28 weeks	14	58.7	32.9	11	60.0	23.8	17.6	(- 46.7, 82.0)
Short-FES-I at baseline	14	13.8	5.3	11	10.7	5.9		
Short-FES-I at 28 weeks	10	11.1	4.0	9	11.9	3.6	−1.2	(−105.7, 103.4)
Mobility assessed with the TUG at baseline	16	41.8	66.3	11	24.3	12.9		
Mobility assessed with the TUG at 28 weeks	12	27.0	19.4	8	31.5	14.6	−9.3	(−461.8, 443.2)
HRQL assessed with QoL-AD at baseline	15	35.0	6.2	12	34.9	8.3		
HRQL assessed with QoL-AD at 28 weeks	11	35.5	5.6	9	34.1	8.0	−0.68	(−141.8, 140.4)
HRQL assessed with QoL-AD Proxy at baseline	15	28.9	6.2	12	32.3	8.5		
HRQL assessed with QoL-AD Proxy at 28 weeks	11	30.4	6.2	9	31.2	8.0	−0.76	(−20.2, 18.7)
Carer burden assessed with ZBI-12 at baseline	18	20.9	10.2	14	18.1	9.2		
Carer burden assessed with ZBI-12 at 28 weeks	12	17.5	13.0	10	17.7	14.7	0.97	(−21.1, 23.1)
HRQL assessed with EQ-5D-5L at baseline	15	0.57	0.20	12	0.65	0.37		
HRQL assessed with EQ-5D-5L at 28 weeks	11	0.69	0.12	10	0.59	0.33	0.10	(−1.2, 1.4)
HRQL assessed with EQ-5D-5L proxy at baseline	18	0.35	0.36	12	0.53	0.32		
HRQL assessed with EQ-5D-5L proxy at 28 weeks	14	0.46	0.26	10	0.59	0.24	−0.08	(−1.2, 1.0)
HRQL assessed with EQ-5D-5L at baseline	18	0.73	0.30	12	0.75	0.23		
HRQL assessed with EQ-5D-5L at 28 weeks	14	0.80	0.13	10	0.61	0.35	0.12	(−0.76, 0.99)

ADL, activities of daily living; DAD, Disability Assessment for Dementia; EQ-5D-5L, EuroQol 5-Dimension 5-Level Instrument; HRQL, health-related quality of life; QoL-AD Proxy, Quality of Life in Alzheimer’s Disease (proxy-rated); QoL-AD, Quality of Life in Alzheimer’s Disease (participant-rated); Short-FES-I, Short Falls Efficacy Scale – International; TUG, Timed Up and Go; ZBI-12, Zarit Burden Interview.

**Table 3:** Frequency of falls assessed using the falls diary

Patient health-related quality of life outcomes	Maintain group			Control group		
	N	Mean	SD	N	Mean	SD
Falls diary at 28 weeks	3	4.7	5.9	4	0.75	1.5
Number of fall days at 28 weeks	8	4.4	3.0	6	1.8	2.2

**Table 4:** Resource use completion

Role	Baseline	3-month follow-up	6-month follow-up
	No. (%)	No. (%)	No. (%)
The person with dementia completed the form	0 (0%)	0 (0%)	0 (0%)
The person with dementia and carer completed the form	6 (20%)	2 (7%)	6 (24%)
The carer completed the form	24 (80%)	25 (93%)	19 (76%)

#### Health economic evaluation

The resource use questionnaire was well completed with a few missing values—all the participants who remained in the study returned the questionnaires at all time points. Completion was primarily by the caregiver (see table 4).

There were no missing data on NHS service use. The most common services were practice nurses (26–60% across time points) and GPs (~40%). Participants also reported using orthopaedics, podiatry, dentistry, cardiology and eye clinics. Engagement with physiotherapists, occupational therapists or rehabilitation support workers outside MAINTAIN was minimal.

All social care service-use questions were completed, though some cost data were missing (n=3–4). About 20% reported home care use at each time point, and 24–33% attended dementia groups. Most received informal support; one in five reported carers missing 1–30 workdays. Travel expenses for carers averaged £50–65, with other out-of-pocket costs including private care, physiotherapy, over-the-counter medications, podiatry and chiropody.

EQ-5D-5L completion was high. At baseline, 27/30 participants responded; one omitted the vertical visual analogue scale (VAS). At 6 months, 21/24 responded; again, one omitted the VAS. Carer proxy EQ-5D-5L was completed for all participants at both time points with no missing data, though carers generally reported more problems than participants (except self-care). Carers’ own EQ-5D-5L was fully completed at both time points.

### Serious adverse events

Across all participating sites, one serious adverse event (SAE) was reported over a cumulative total of 8195 patient days (0.0001 SAEs per patient day). The reported SAE involved a participant who fell outside on the pavement, resulting in a hand injury requiring hospital assessment and outpatient follow-up. The event was classified as mild in severity, unrelated to the study intervention and had a favourable outcome with full resolution.

## Discussion

This pilot study demonstrated the feasibility of delivering the MAINTAIN intervention to people living with dementia after a fall and of conducting a randomised trial in this population. Although the pilot did not reach the planned sample size of 60 dyads, recruitment, retention and attendance met our prespecified stop-go criteria. Of 37 eligible participants, 31 consented (84%), exceeding the ≥40% recruitment threshold and 25 of 31 (81%) provided 28-week outcome data, surpassing the ≥70% retention criterion. Attendance in the intervention arm also met the stop-go criterion, with most participants attending at least 60% of planned sessions. Thus, despite fewer eligible participants than anticipated, the core trial processes functioned as intended. Together, these findings demonstrate strong participant engagement and indicate that structured, home-based exercise and rehabilitation programmes are acceptable to people with dementia and their caregivers. This study benefited from the use of a cluster randomised controlled design, enabling evaluation of implementation processes in real-world community and health service settings.

Economic and quality-of-life data were collected successfully, and outcome measures were acceptable to participants and carers. However, cluster randomisation before participant enrolment may have introduced baseline imbalance and selection bias. Falls data were incomplete due to low diary return rates and reporting difficulties, highlighting methodological challenges common in dementia research. The study sample was predominantly white and reliant on unpaid carers, which may limit generalisability. Because this was a cluster randomised trial, research staff were necessarily aware of site allocation prior to recruitment and baseline assessments. As a result, baseline outcome measurements could not be blinded, introducing a risk of observer bias. This limitation is inherent to service-level cluster designs, where allocation determines site procedures.

Engagement levels were comparable to, or higher than, those observed in other dementia rehabilitation trials. In the Dementia And Physical Activity trial, more than 65% of participants met the compliance threshold,^11^ and in the Promoting Activity, Independence and Stability in Early Dementia and mild cognitive impairment trial, approximately 80% were retained at 12 months.^34^ Consistent with these findings, the current study supports the acceptability of exercise-based interventions for people with dementia and their families. As in other trials, recruitment proved variable; community and mental health sites with established dementia caseloads performed well, whereas acute hospital sites struggled, reflecting the known challenges of recruitment infrastructure and staff availability.^35^

The embedded framework for economic data collection was successfully implemented and assessed to be both feasible and useful. While the data collected in the study do not inform cost or cost effectiveness, the updated forms will be used in a future full RCT of the intervention for cost and cost effectiveness analysis. The feasibility of economic data collection, including high completion of the EQ-5D-5L by both participants and proxies, demonstrates that quality-of-life assessment can be incorporated effectively in this population. However, discrepancies between self-reports and proxy-reports underline the importance of predefining the primary source of quality-adjusted life year estimation.^36^,^38^ The challenges in falls data collection highlight the need for pragmatic alternatives, such as telephone follow-up or digital monitoring, to improve data accuracy.^39^

The MAINTAIN intervention aligns with current policy priorities promoting rehabilitation and independence in dementia care and could contribute to post-fall recovery pathways if proven effective at scale. Future trials should adopt an individually randomised design to minimise baseline imbalances and reduce the risk of selection bias. Recruitment efforts should focus on community and mental health settings, with hospital sites supported by dedicated research staff. Refinements to falls data collection methods and clearer specification of self versus proxy quality-of-life reporting will strengthen outcome validity. With these adaptations, a definitive trial could provide robust evidence on the clinical and cost-effectiveness of the MAINTAIN intervention and inform national strategies for post-fall rehabilitation in people living with dementia.

## Supplementary material

10.1136/bmjopen-2025-112336 online supplemental file 1

## Data Availability

Data are available upon reasonable request.
