# Supplementary material for: Feasibility of the MAINTAIN intervention to support independence after a fall for people with dementia: a pilot cluster randomised controlled trial in participants’ own homes
Source: BMJ Open. 2026 Feb 10;16(2):e112336. doi: 10.1136/bmjopen-2025-112336 (PMC12911774; doi:10.1136/bmjopen-2025-112336)
Supplement: online supplemental file 1 [file bmjopen-16-2-s001.docx]

**Appendix 1.** Consolidated Standards of Reporting Trials (CONSORT) checklist.


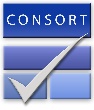
CONSORT 2010 checklist of information to include when reporting a pilot or feasibility trial*

| Section/Topic | Item No | Checklist item | Reported on page No |
| --- | --- | --- | --- |
| Title and abstract | | | |
|  | 1a | Identification as a pilot or feasibility randomised trial in the title | 1 |
|  | 1b | Structured summary of pilot trial design, methods, results, and conclusions (for specific guidance see CONSORT abstract extension for pilot trials) | 1 |
| Introduction | | | |
| Background and objectives | 2a | Scientific background and explanation of rationale for future definitive trial, and reasons for randomised pilot trial | 3-5 |
|  | 2b | Specific objectives or research questions for pilot trial | 5 |
| Methods | | | |
| Trial design | 3a | Description of pilot trial design (such as parallel, factorial) including allocation ratio | 6-7 |
|  | 3b | Important changes to methods after pilot trial commencement (such as eligibility criteria), with reasons | N/A |
| Participants | 4a | Eligibility criteria for participants | 8-9 |
|  | 4b | Settings and locations where the data were collected | 8 |
|  | 4c | How participants were identified and consented | 8 |
| Interventions | 5 | The interventions for each group with sufficient details to allow replication, including how and when they were actually administered | 6 – reference to protocol |
| Outcomes | 6a | Completely defined prespecified assessments or measurements to address each pilot trial objective specified in 2b, including how and when they were assessed | 6 – reference to protocol |
|  | 6b | Any changes to pilot trial assessments or measurements after the pilot trial commenced, with reasons | N/A |
|  | 6c | If applicable, prespecified criteria used to judge whether, or how, to proceed with future definitive trial | 17 |
| Sample size | 7a | Rationale for numbers in the pilot trial | 13-14 |
|  | 7b | When applicable, explanation of any interim analyses and stopping guidelines | N/A |
| **Randomisation:** |  |  |  |
| Sequence  generation | 8a | Method used to generate the random allocation sequence | 6-7 |
|  | 8b | Type of randomisation(s); details of any restriction (such as blocking and block size) | 6-7 |
| Allocation  concealment  mechanism | 9 | Mechanism used to implement the random allocation sequence (such as sequentially numbered containers), describing any steps taken to conceal the sequence until interventions were assigned | 6-7 |
| Implementation | 10 | Who generated the random allocation sequence, who enrolled participants, and who assigned participants to interventions | 6-7 |
| Blinding | 11a | If done, who was blinded after assignment to interventions (for example, participants, care providers, those assessing outcomes) and how | 6-7 |
|  | 11b | If relevant, description of the similarity of interventions | N/A |
| Statistical methods | 12 | Methods used to address each pilot trial objective whether qualitative or quantitative | 14-16 |
|  |  |  |  |
| Results | | | |
| Participant flow (a diagram is strongly recommended) | 13a | For each group, the numbers of participants who were approached and/or assessed for eligibility, randomly assigned, received intended treatment, and were assessed for each objective | Figure 1 |
|  | 13b | For each group, losses and exclusions after randomisation, together with reasons | Figure 1 |
| Recruitment | 14a | Dates defining the periods of recruitment and follow-up |  |
|  | 14b | Why the pilot trial ended or was stopped | N/A |
| Baseline data | 15 | A table showing baseline demographic and clinical characteristics for each group | Table 2 |
| Numbers analysed | 16 | For each objective, number of participants (denominator) included in each analysis. If relevant, these numbers  should be by randomised group | 18-24 |
| Outcomes and estimation | 17 | For each objective, results including expressions of uncertainty (such as 95% confidence interval) for any  estimates. If relevant, these results should be by randomised group | 18-24 |
| Ancillary analyses | 18 | Results of any other analyses performed that could be used to inform the future definitive trial | 25-27 |
| Harms | 19 | All important harms or unintended effects in each group (for specific guidance see CONSORT for harms) | 27 |
|  | 19a | If relevant, other important unintended consequences | N/A |
| Discussion | | | |
| Limitations | 20 | Pilot trial limitations, addressing sources of potential bias and remaining uncertainty about feasibility | 31 |
| Generalisability | 21 | Generalisability (applicability) of pilot trial methods and findings to future definitive trial and other studies | 27-32 |
| Interpretation | 22 | Interpretation consistent with pilot trial objectives and findings, balancing potential benefits and harms, and  considering other relevant evidence | 27-32 |
|  | 22a | Implications for progression from pilot to future definitive trial, including any proposed amendments | 27-32 |
| Other information | | |  |
| Registration | 23 | Registration number for pilot trial and name of trial registry | 6 |
| Protocol | 24 | Where the pilot trial protocol can be accessed, if available | 6 |
| Funding | 25 | Sources of funding and other support (such as supply of drugs), role of funders | 33 |
|  | 26 | Ethical approval or approval by research review committee, confirmed with reference number | 6 |

Citation: Eldridge SM, Chan CL, Campbell MJ, Bond CM, Hopewell S, Thabane L, et al. CONSORT 2010 statement: extension to randomised pilot and feasibility trials. BMJ. 2016;355. This is an Open Access article distributed in accordance with the terms of the Creative Commons Attribution (CC BY 3.0) license (<http://creativecommons.org/licenses/by/3.0/>), which permits others to distribute, remix, adapt and build upon this work, for commercial use, provided the original work is properly cited.

*We strongly recommend reading this statement in conjunction with the CONSORT 2010, extension to randomised pilot and feasibility trials, Explanation and Elaboration for important clarifications on all the items. If relevant, we also recommend reading CONSORT extensions for cluster randomised trials, non-inferiority and equivalence trials, non-pharmacological treatments, herbal interventions, and pragmatic trials. Additional extensions are forthcoming: for those and for up-to-date references relevant to this checklist, see [www.consort-statement.org](http://www.consort-statement.org).

**Appendix 2. MAINTAIN Statistical Analysis Plan**
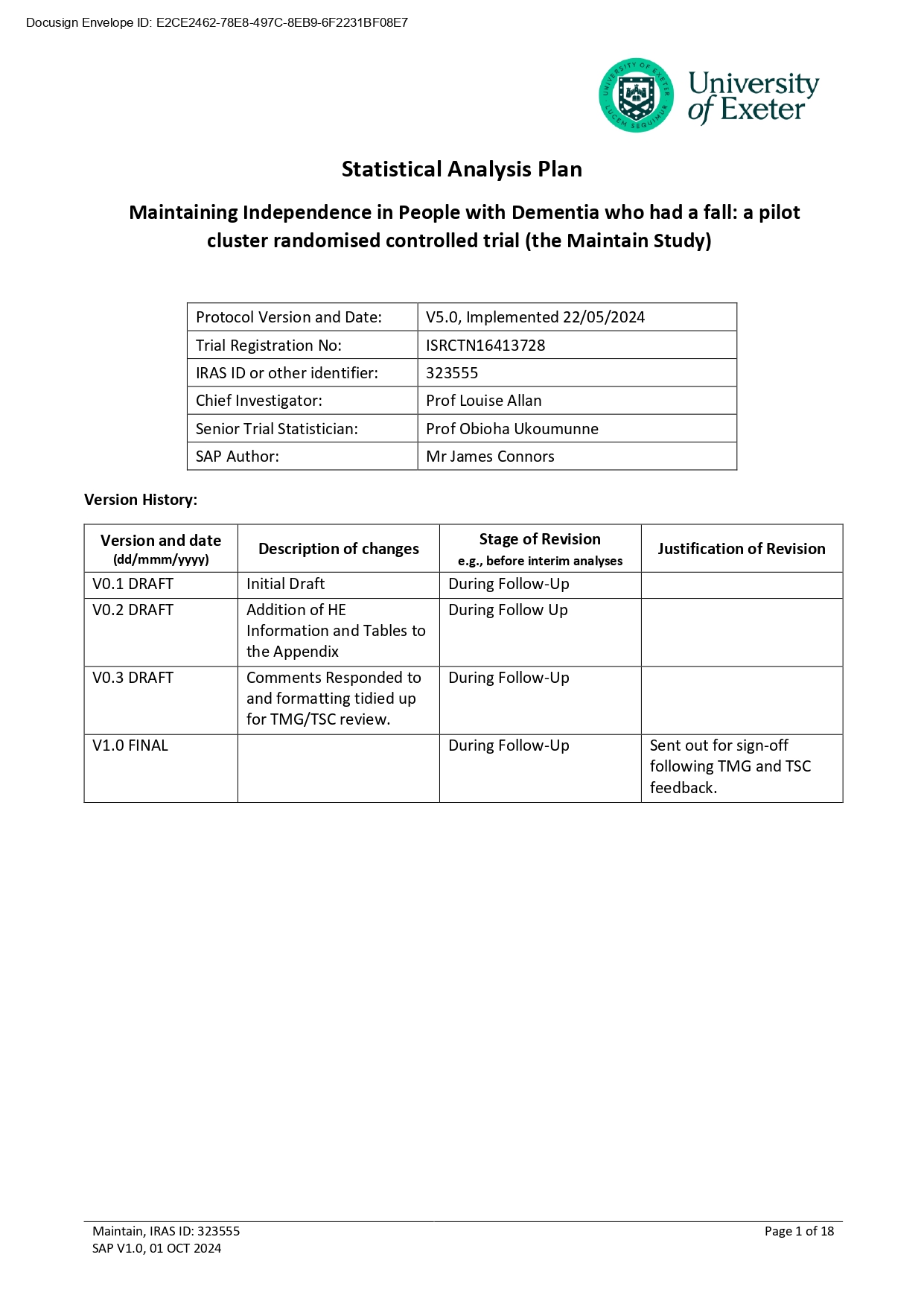

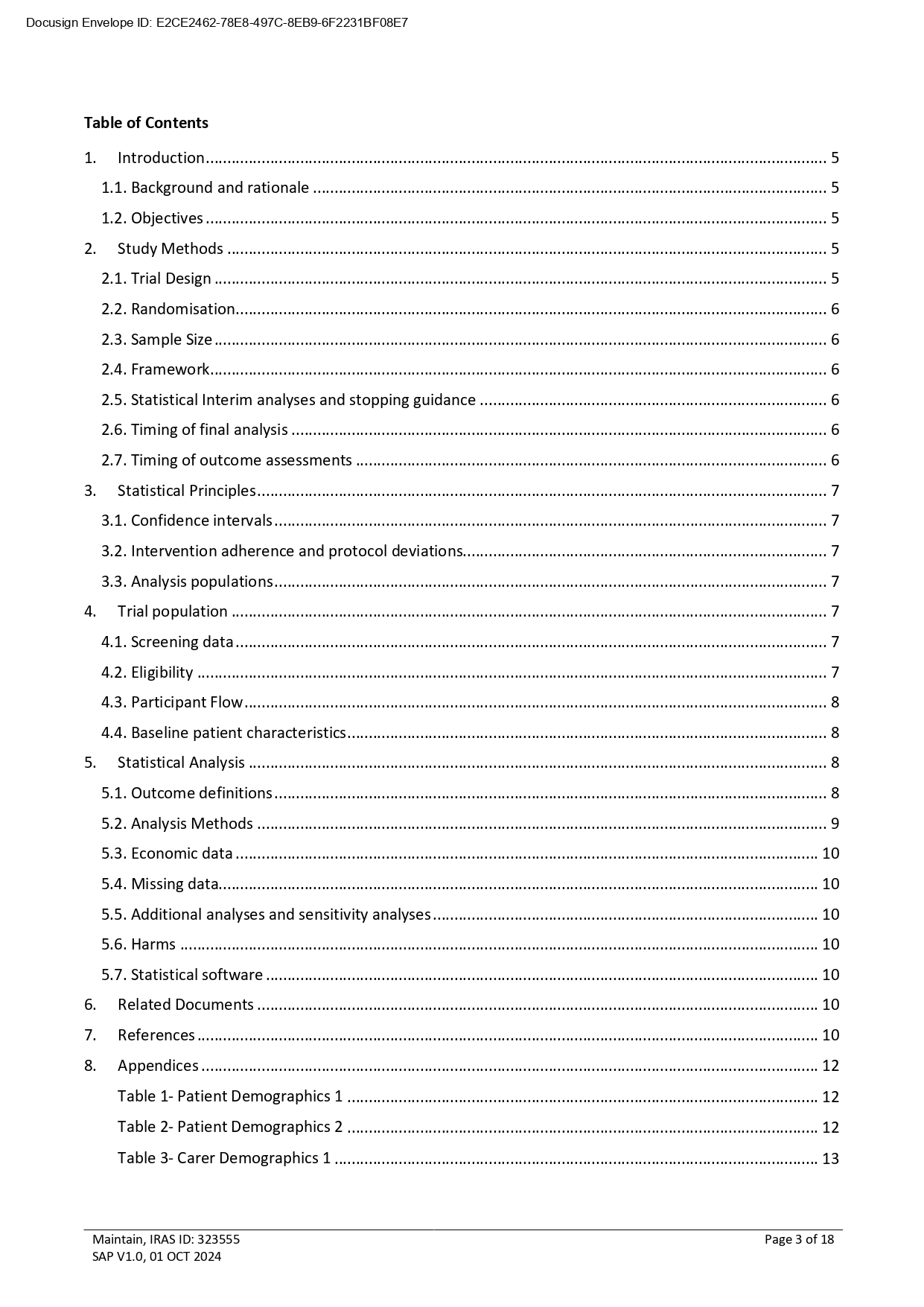

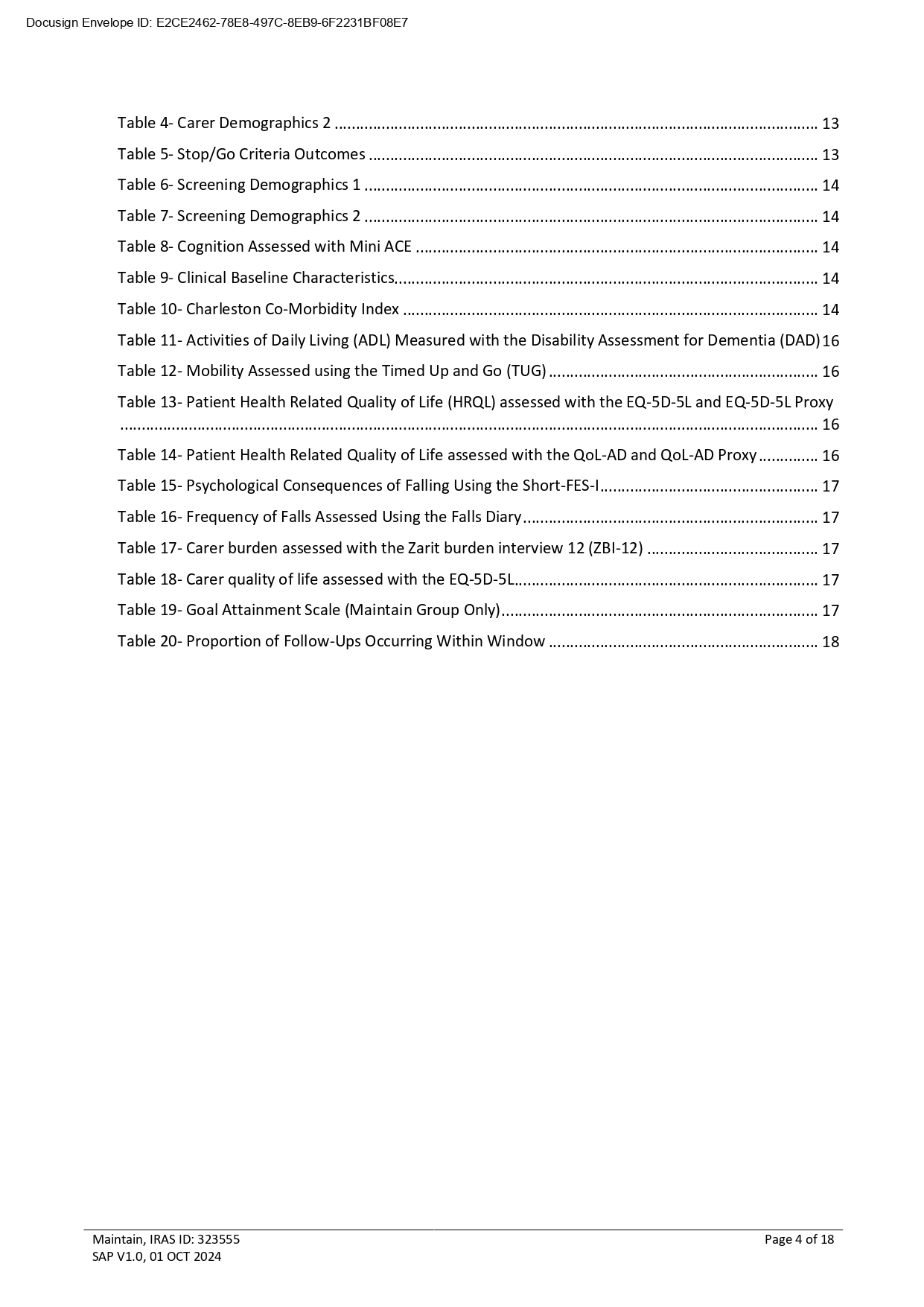

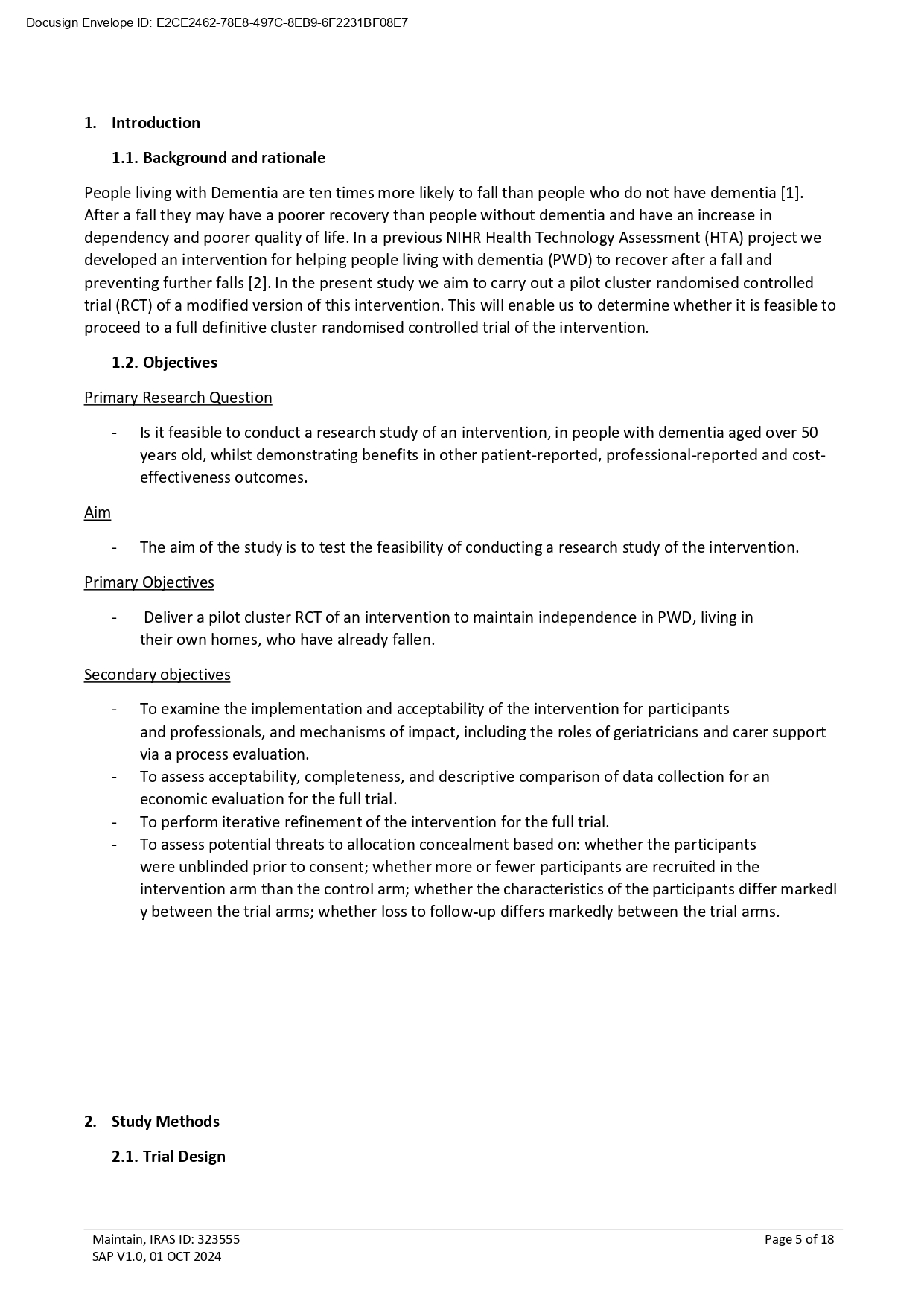

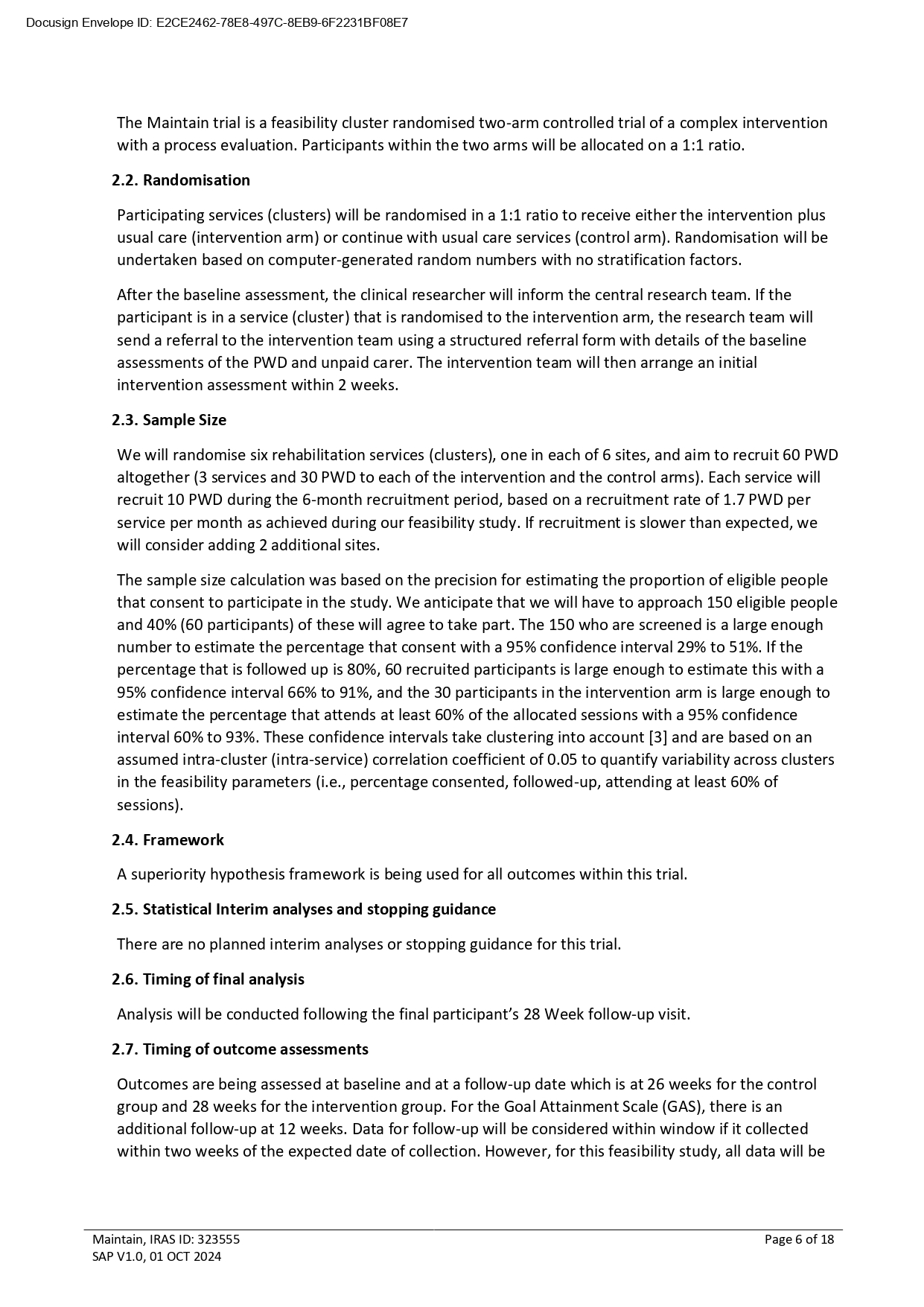

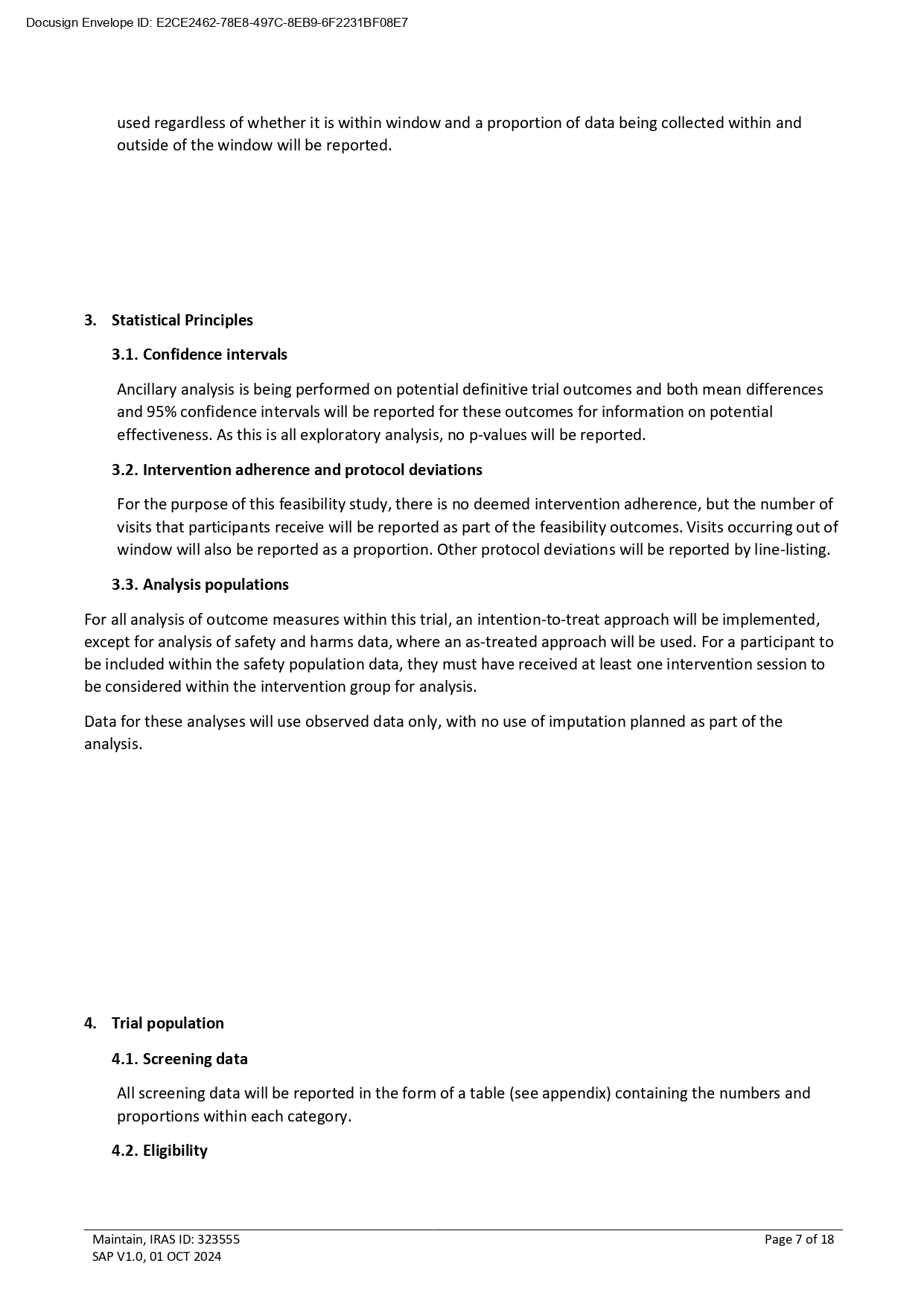

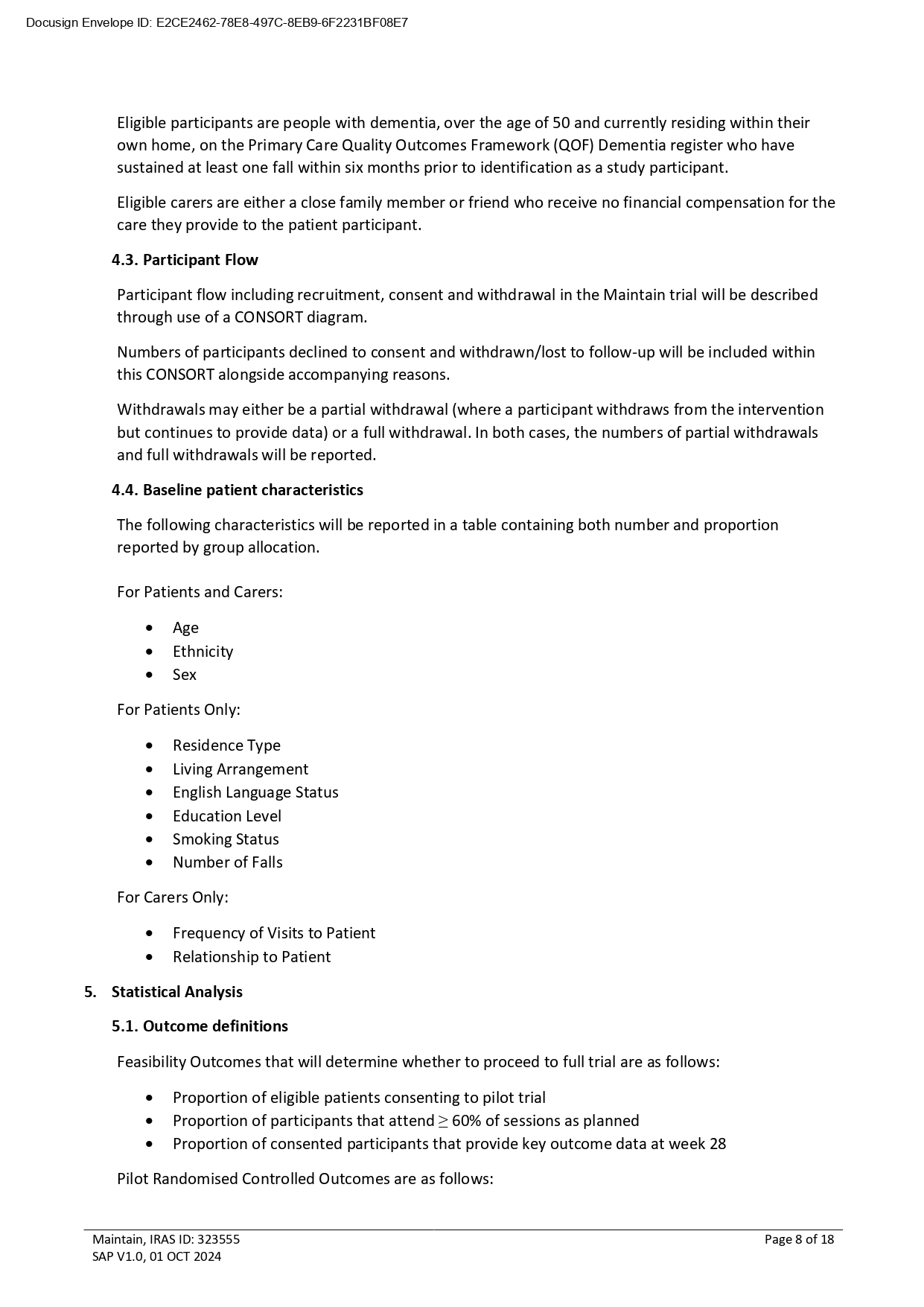

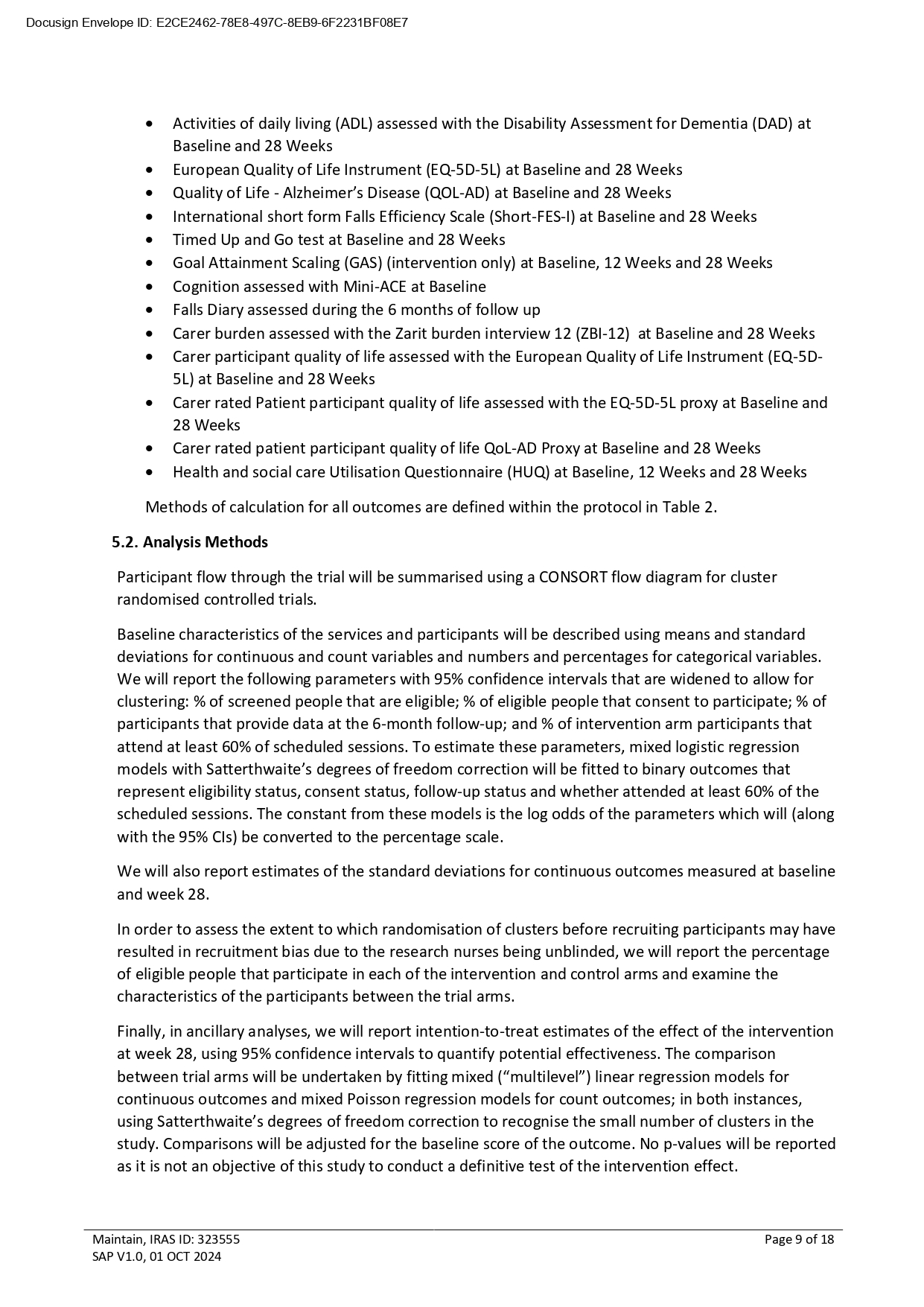

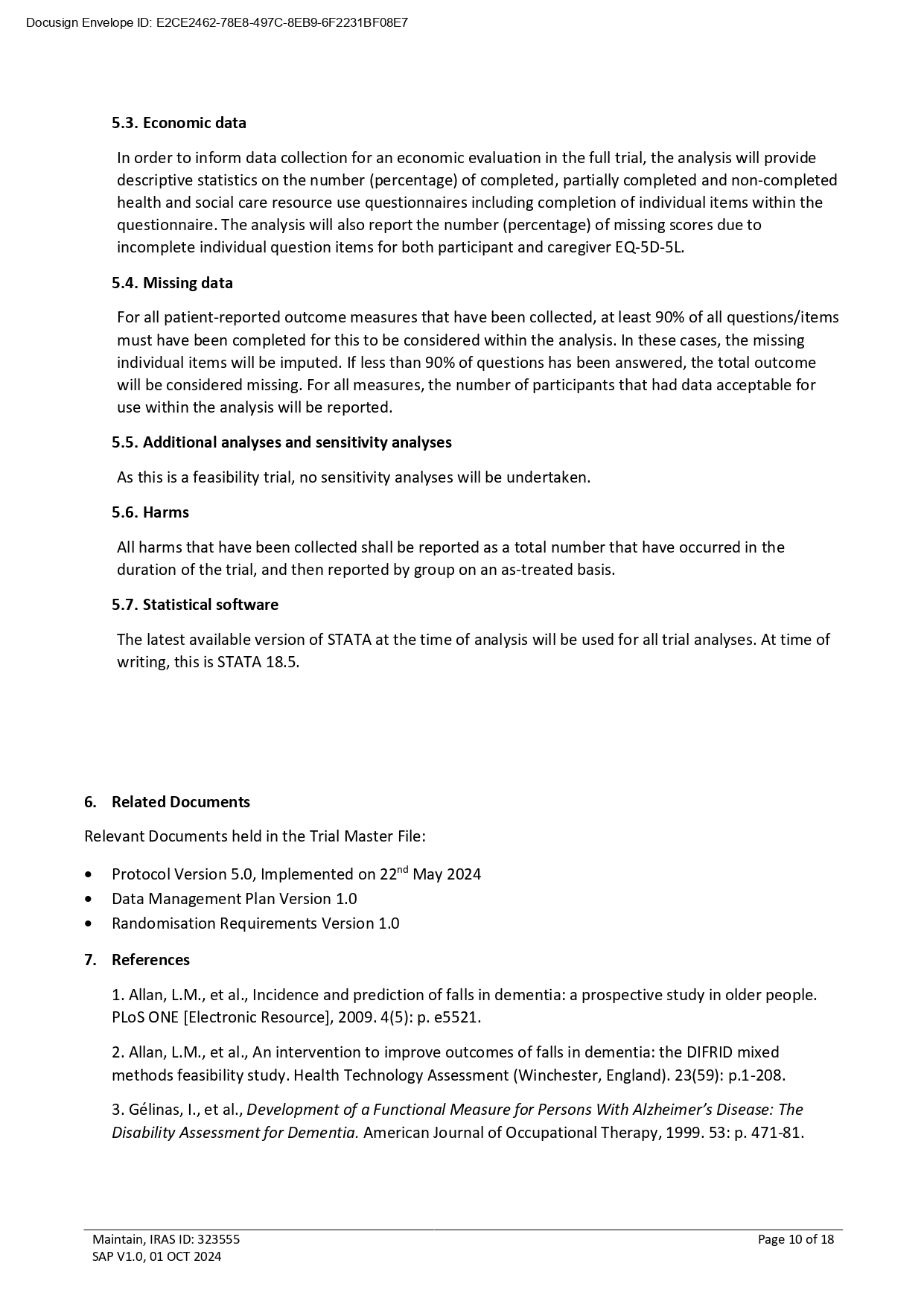

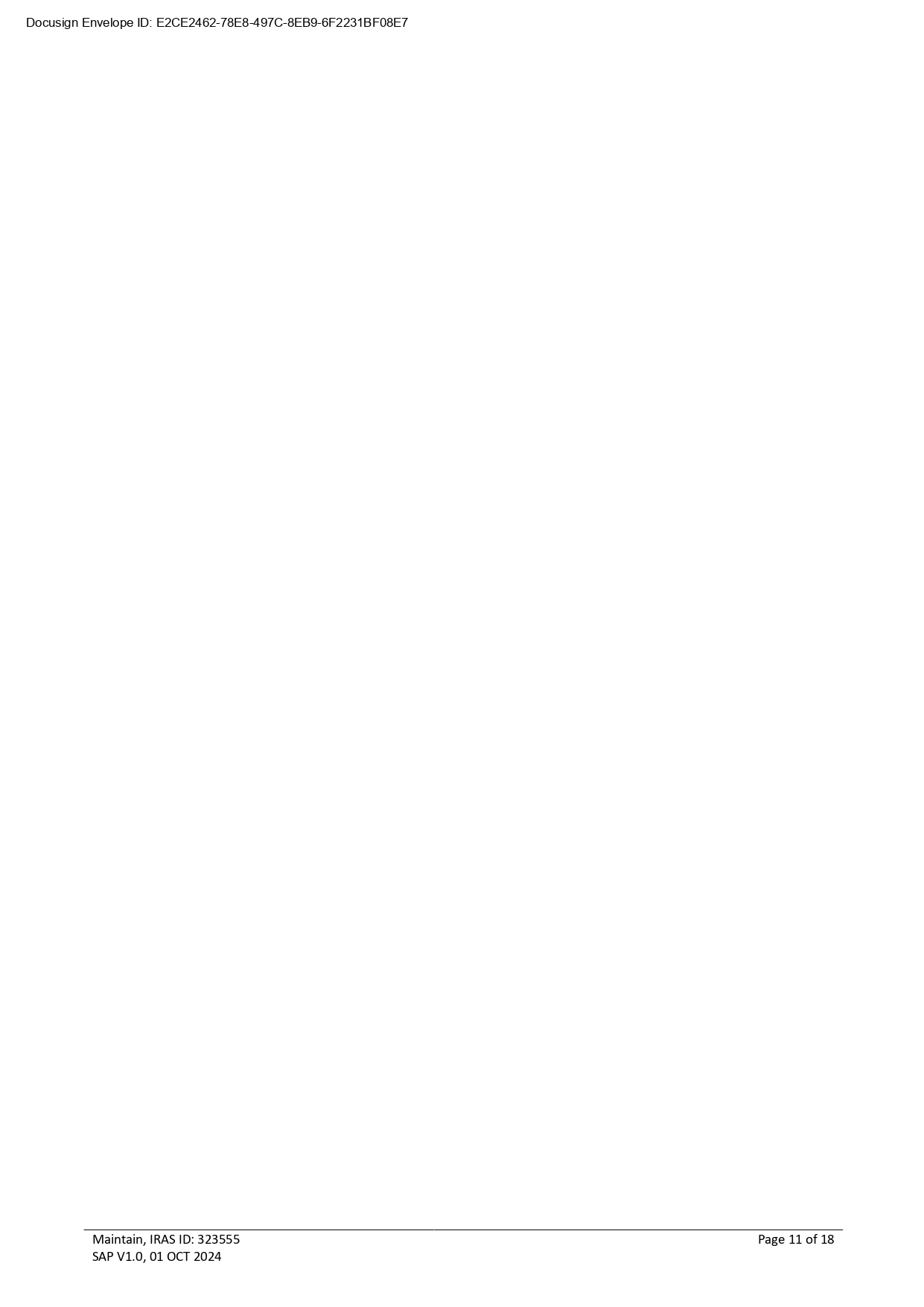

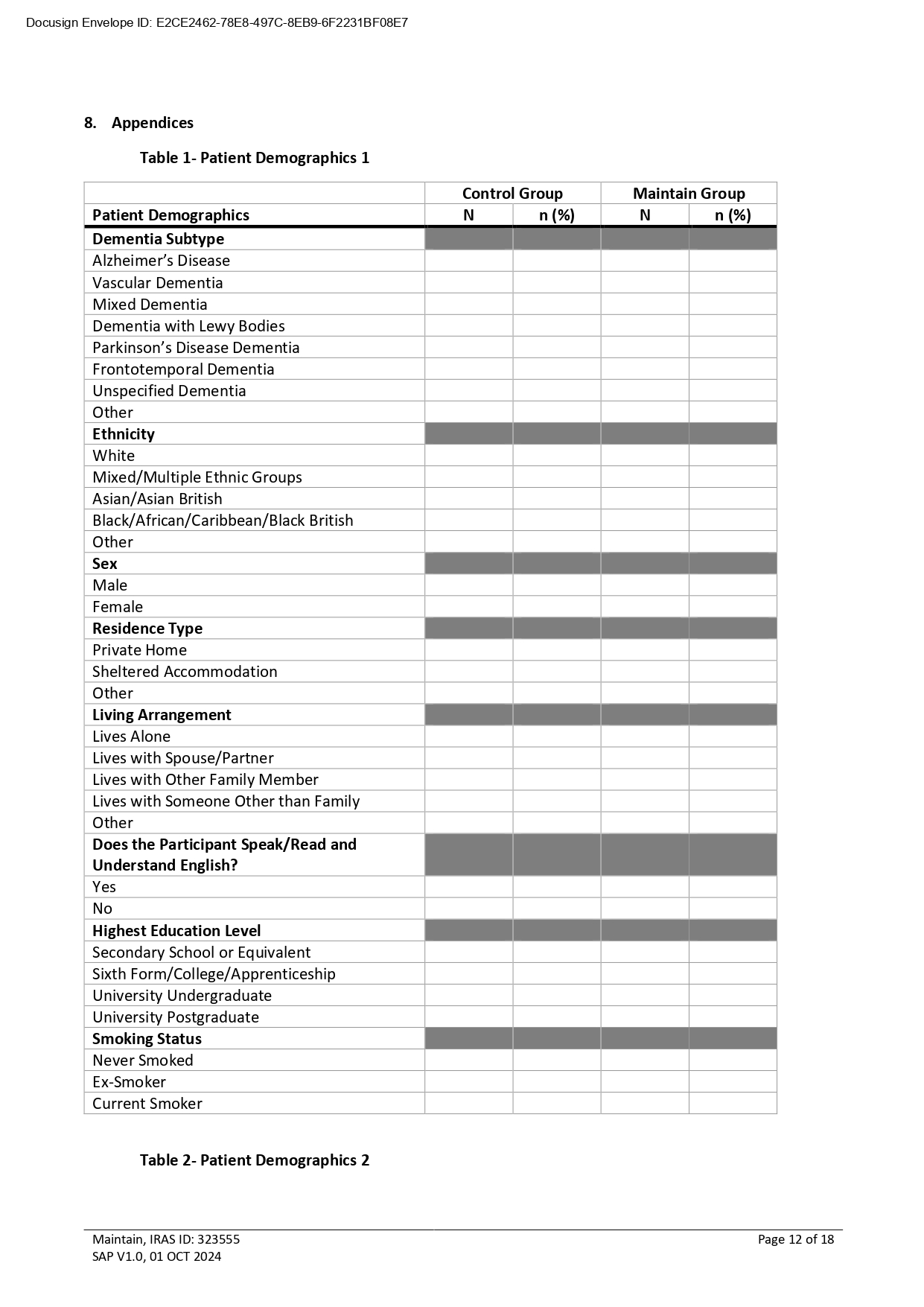

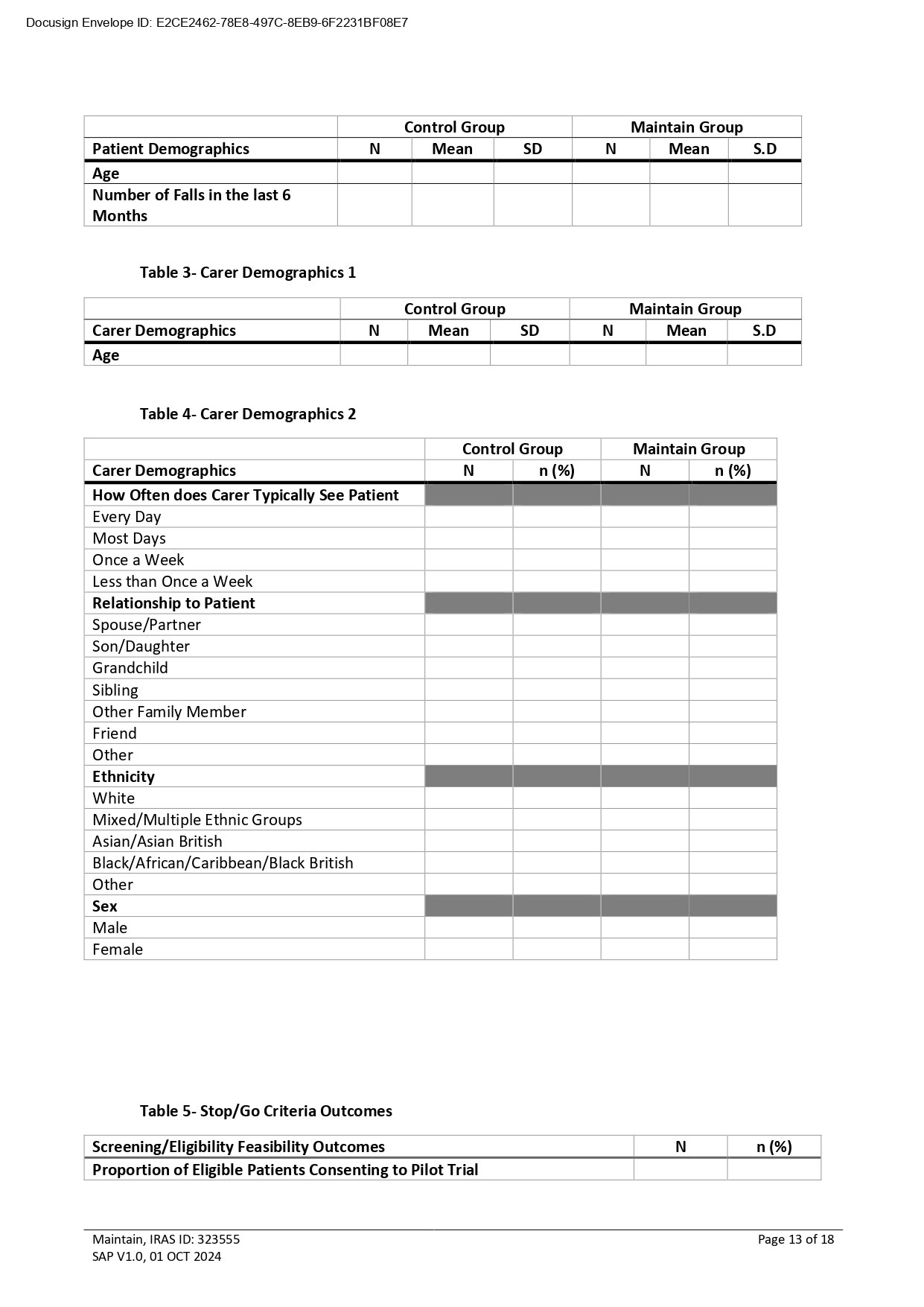

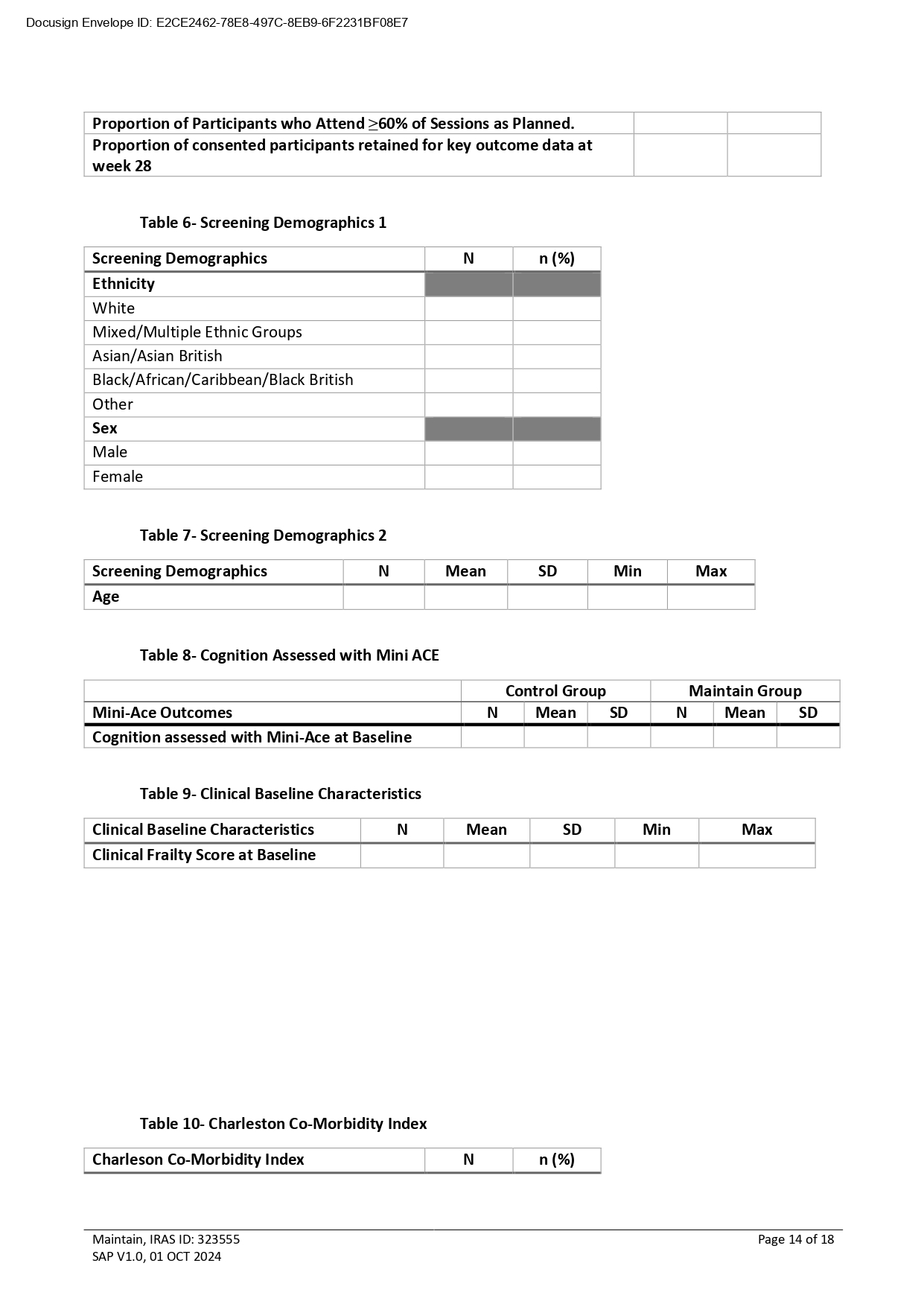

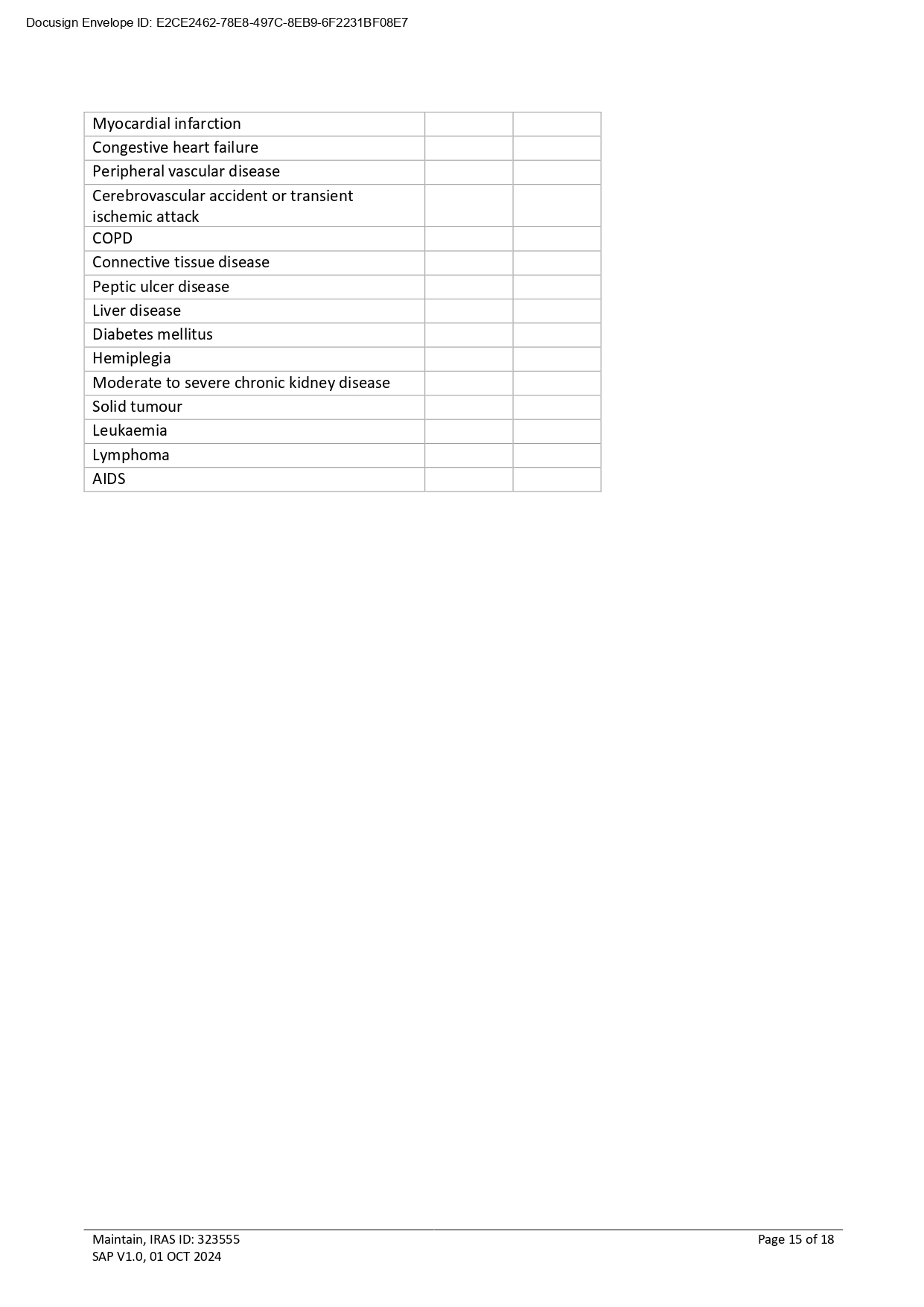

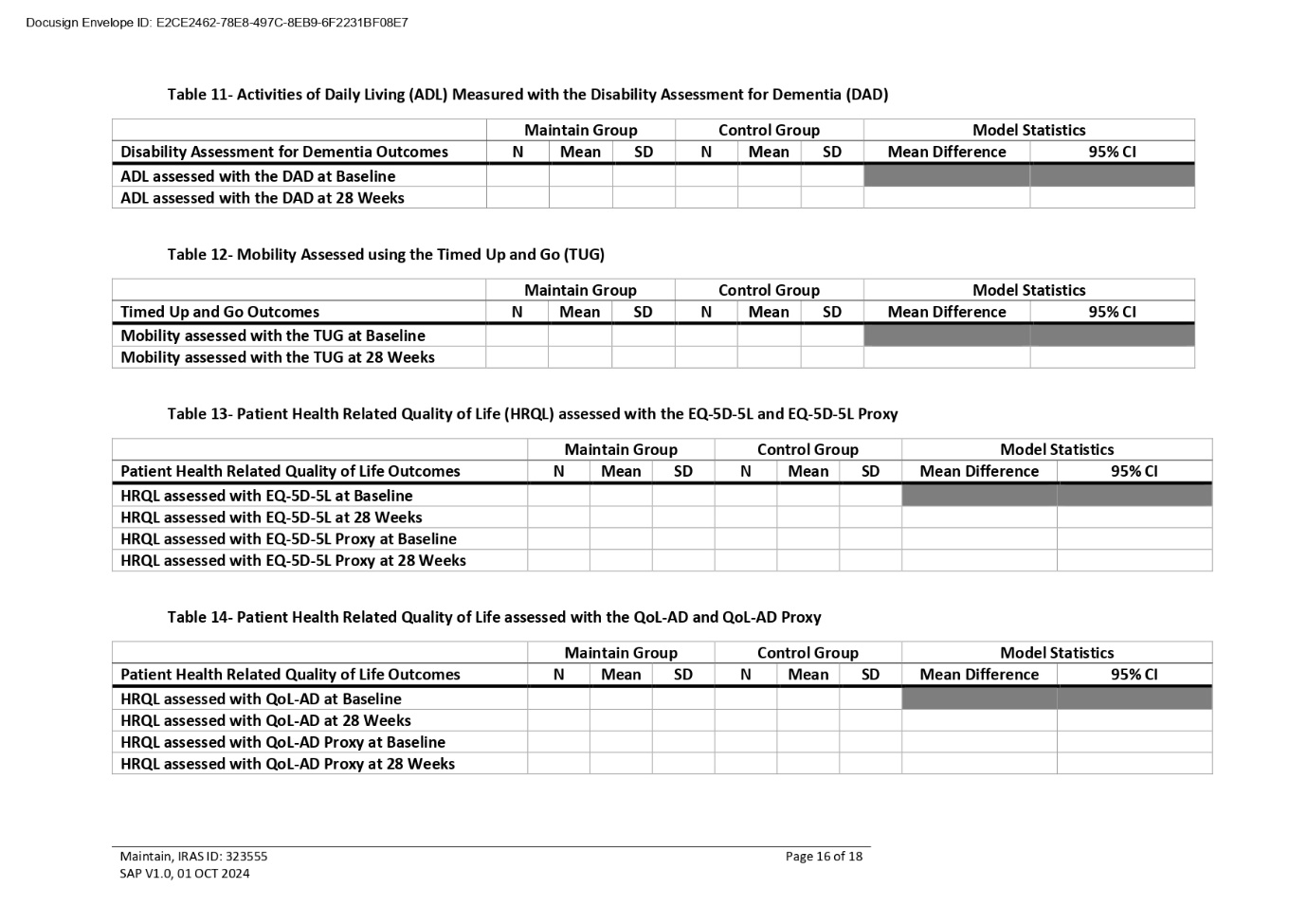

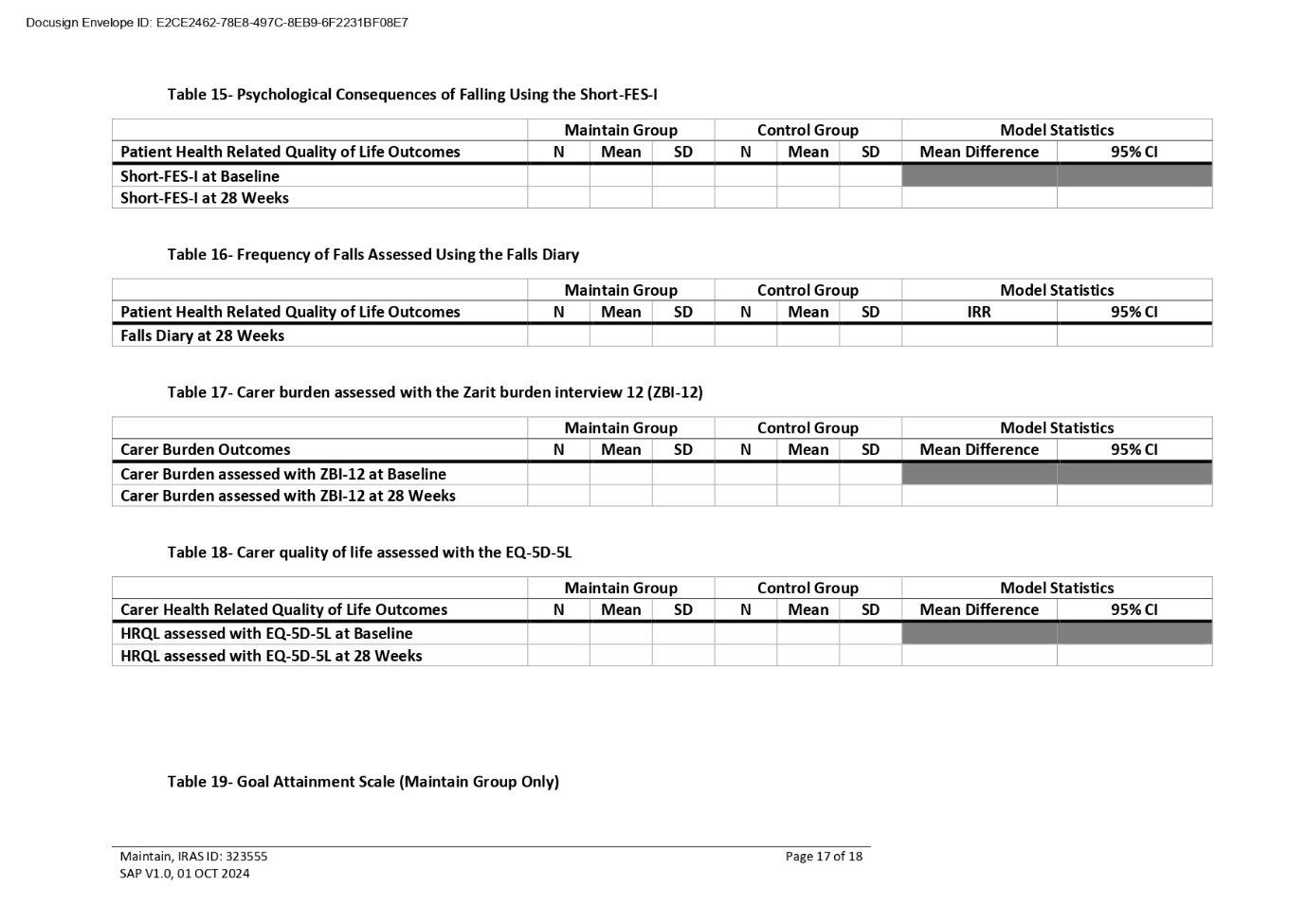


**Appendix 3. Site characteristics for the MAINTAIN pilot trial.**

**Site 1 – Intervention site**

Site 1 is embedded in an NHS Trust that provides a full range of mental health services, as well as support for people with learning disabilities and individuals with autism. The team delivering the intervention were an NIHR Research Delivery Team who are a member of the local Clinical Research Network (CRN). The team operates within the Medical Directorate, overseeing clinical research initiatives focused on mental health throughout the county. They administer Research Governance for ongoing studies, with several being affiliated with the NIHR but also supporting alternatively funded projects. At any given time, the team will concurrently manage multiple research projects, each at various stages of progress.

The team comprises a senior research nurse, three research nurses, and four clinical research practitioners. The MAINTAIN team consisted of a band 7 Parkinson's specialist physiotherapist, a band 7 occupational therapist (OT), and three rehabilitation support workers (RSW). One RSW was a band six mental health nurse and two RSWs were band four. Despite the clinical backgrounds of the team members, their research roles are distinct and independent. They do not have clinical responsibilities and must therefore collaborate with clinicians or internal/external agencies for recruitment purposes. The local site Principal Investigator is from within the Trust and other team members have worked for the Trust for many years so they have established relationships with colleagues who may be able to recruit. They also have access to Join Dementia Research and Age UK (e.g. I Forget Service). Several referrals have come from Dementia Support Workers who are employed by the local Community Mental Health Team. One of the RSWs used to work in an NHS Memory Clinic and does a lot of volunteering with dementia cafes and this has boosted recruitment rates.

The team held weekly multidisciplinary meetings and formal supervision sessions as well as having a therapy support WhatsApp group.

**Site 2 – Intervention site**

Site 2 operates as an independent charity that facilitates research and offers assistance to individuals affected by dementia and other conditions associated with older age, as well as their families and caregivers. The clinical researcher and RSW were both Site 2 employees and were both research psychologists (also known as assistant psychologists in the NHS). Site 2 employees did not have their usual clinical duties reduced when they were running MAINTAIN. Site 2 employed the services of an OT and a physiotherapist from a small private practice offering assessment and treatments in people’s homes. The physiotherapist and OT would do a joint home assessment and then discuss the participant's goals and activity programme afterwards. The site did not hold an MDT with the RSW and no supervision was offered to the RSW. Participants were recruited by calling previous service users to determine eligibility and by referrals from clinicians working within the site (e.g. doctors from the memory service).

**Site 3 – Intervention site**

Site 3 was embedded within an NHS Foundation Trust that provides comprehensive NHS services throughout a person’s life, encompassing physical and mental health care, delivered in various settings including general practices, hospitals, the community, and patients' homes. The majority of services are delivered within the community, focusing on treating individuals in their homes or at nearby clinics. When community care is not feasible, the Trust offers a range of facilities for treatment in hospital or residential environments. Additionally, healthcare services are provided in prisons across the local area.

The Clinical Researcher was a Research Practitioner within the local NHS-based Research Office that serves a partnership of local NHS Trusts, primary care organisations, social care services, and various settings outside the NHS. As a research office, the team provides various services to support studies from initial concept to delivery. This includes processing sponsorship requests for staff employed by partner trusts and preparing the necessary review contracts for site participation. Dedicated teams support research in primary care and settings outside the NHS, coordinating study set-up, site selection, and delivery. The delivery teams assist trusts with engaging services, identifying patients, and obtaining informed consent. The research office has formal partnerships with several local NHS Trusts that provide mental health and community services. Collaboration also extends to various allied academic institutions and other organisations.

Participants were recruited through a Rapid Access Service, which encompassed the Urgent Community Response, Virtual Ward, and Discharge to Assess pathways. The Rapid Access Service offers three distinct pathways focused on admission avoidance and early discharge. The multidisciplinary team includes Advanced Nurse Practitioners, Paramedics, Occupational Therapists, Physiotherapists, Doctors, Nursing Associates, and Senior Healthcare Assistants. The primary aim of the service is to support individuals with immediate health or functional needs who might otherwise require hospitalisation. It is part of a broader range of intermediate care services providing therapy and rehabilitation in community settings for adults in the local community who have conditions such as urinary tract infections, chest infections, heart failure, dehydration, frailty, falls, and hypertension.

Screening of potential participants was conducted by the Virtual Ward clinical team, who then referred eligible individuals to the Clinical Researcher based at the local NHS Research Office. Despite these established processes, recruitment at this site proved challenging, with no participants ultimately enrolled. Subsequent interview data with the Clinical Researcher highlighted several potential contributing factors to these recruitment difficulties. These included the Clinical Researcher not being physically integrated within the recruiting team, the clinical acuity of the service users being incompatible with an intervention focused on functional maintenance, and the competing demands on clinicians' time, where clinical priorities necessarily took precedence over research activities.

**Site 4 – Treatment as usual site**

Site 4 is part of an NHS Foundation Trust that provides mental health, learning disabilities, and neuro-rehabilitation services. The team are part of the NIHR Portfolio Research Delivery Team that, in a typical year, hosts between 50 to 60 large-scale NIHR projects. As a vital component of the regional research infrastructure, the site serves as the host for the local NIHR Applied Research Collaboration.

The site maintains two dedicated research registers that allow interested participants to join a mailing list for updates about relevant research opportunities. Everyone who goes through the local Memory Clinic has the option to sign up for the register. The Clinical Researcher was a Mental Health Nurse and had no clinical duties.

**Site 5 – Treatment as usual site**

Site 5 is embedded within an NHS Trust that provides community and mental health services. The Trust collaborates with schools, local hospitals, GP practices, social services, and other local authority departments such as housing and education. Additionally, partnerships with voluntary organisations and local community groups are established to achieve goals and ensure that care recipients are treated to the highest possible standard. Care and support are delivered through three directorates, (i) mental health services, (ii) families, young people, children’s, learning disability and autism services, and (iii) community health services. The Trust supports a wide range of research activities. At any given time, it backs several NIHR CRN portfolio studies, with participants drawn from various service areas and staff groups. This research is facilitated by the CRN-funded Research Delivery Team and is authorised through the Trust's R&D Office.

The two MAINTAIN Clinical Researchers support the CRN dementia portfolio. Participants were referred through GP surgeries doing database searches on their behalf, and also, via the falls clinic, and also, via the mental health for older adults team, including the memory service. The Research Delivery Team operates independently, initiating contact with clinical teams and collaborating on research activities, while maintaining a distinct separation from the clinical services themselves. The CRN staff all have research-specific areas including dementia and the Clinical Researchers had several years of experience in research.

**Site 6 – Treatment and usual site**

Site 6 operates within one of England's largest NHS Foundation Trust teaching hospitals. This multi-hospital Trust provides comprehensive healthcare services to a demographically diverse population. The Trust maintains strong academic partnerships to facilitate clinical research, with research activities fully integrated within clinical services to support evidence-based practice implementation. Participant screening was conducted by a consultant from the Older People's team.

**Appendix 4. Schedule of events for the MAINTAIN intervention and control arms.**

**Appendix 5. Success criteria and barriers to success for the MAINTAIN trial.**

| Success Criteria for MAINTAIN Trial | Definite go (“green light”) | Definite stop (“red light”) |
| --- | --- | --- |
| Recruitment | ≥40% of eligible patients consenting to the pilot trial | <10% of eligible participants consenting to the pilot trial |
| Attendance | ≥80% of participants attending ≥60% of planned sessions | <30% of participants attending ≥60% of planned sessions in each intervention arm |
| Retention | Retention of ≥70% of consented participants for key outcome data at 28 weeks | Retention of <50% of consented participants for the provision of key outcome data at 28 weeks |
| Acceptability/Fidelity | Qualitative feedback indicating that the intervention is perceived as acceptable to both participants and professionals | Process data from participants and professionals indicating low fidelity in intervention procedures (content, frequency, duration and quality), or the intervention being deemed infeasible to deliver |
