# Supplementary material for: Feasibility of the MAINTAIN intervention to support independence after a fall for people with dementia: a pilot cluster randomised controlled trial in participants’ own homes
Source: BMJ Open. 2026 Feb 10;16(2):e112336. doi: 10.1136/bmjopen-2025-112336 (PMC12911774; doi:10.1136/bmjopen-2025-112336)
Supplement: Supplementary file 2 [file bmjopen-2025-112336.reviewer_comments.pdf]

PEER REVIEW HISTORY

BMJ Open publishes all reviews undertaken for accepted manuscripts. Reviewers are asked to complete a checklist review form and are provided with free text boxes to elaborate on their assessment. These free text comments are reproduced below.

ARTICLE DETAILS

Title (Provisional)

Feasibility of the MAINTAIN intervention to support independence after a fall for people with dementia: a pilot cluster randomised controlled trial in participants' own homes.

Authors

Greene, Leanne; Duff, Carol; Morgan-Trimmer, Sarah; Sharma, Ashima; Connors, James; Hulme, Claire; Ukoumunne, Obioha C; Barber, Robert; Bingham, Alison; Conroy, Simon; Fox, Chris; Goodwin, Victoria; Gordon, Adam L; Hall, Abigail J.; Harwood, Rowan H; Jackson, Thomas; Litherland, Rachael; Parry, Steve W; Whale, Bethany; Allan, Louise

---

VERSION 1 - REVIEW

**Reviewer**	**1**
**Name**	**Chenoweth, Lynn L**
**Affiliation**	**University of New South Wales, School of Psychiatry**
**Date**	**26-Oct-2025**
**COI**	**None**

This pilot trial has been conducted according to the published protocol, with the findings aligning closely with the study aims and objectives. The limitations arising with study recruitment, randomization and data collection have been clearly identified, and suitable remedial strategies/processes identified for a future full trial.

There are a few issues requiring further details, as follows:

Page 7, line 47. Explain what 'PPIE panel' refers to. If this is an expert advisory panel, provide brief details in who was included in the panel and how they were recruited.

Page 7, lines 25-26. Provide further details on who the health professionals were who identified, recruited and consented study participants. Were these members of the research team, employed research assistants, or health professionals working in each of the services the potential participants attended? If participant recruitment and consent was undertaken by health professionals providing services to these clients would pose risk of recruitment bias as well as potential coercion, how was this managed?

Page 13, line 11. Insert an additional bracket at the end of (CI:68% to 94&).

**Reviewer**	**2**
**Name**	**Lichtwarck, Bjørn**
**Affiliation**	**Innlandet Hospital Trust**
**Date**	**10-Nov-2025**
**COI**	**None**

Dear authors,

It is commendable that you have conducted a feasibility study prior to undertaking a definitive randomized controlled trial. The topic you address is important, particularly in a field where there is a scarcity of intervention studies. Conducting such preliminary work is essential to ensure methodological robustness and to optimize the design of future large-scale trials.

Nevertheless, there are several limitations and areas that require clarification or further development in the current manuscript. Below, I outline my main comments and suggestions for improvement

Firstly: Abbreviations must be written out in full the first time they are mentioned. Please check the manuscript carefully for such abbreviations.since there are some abbreviations not written out in full text.

Title:

The title should also reflect design of a study, e.g., Maintaining independence after a fall: a pilot cluster randomised trial of the MAINTAIN intervention for people with dementia

The abstract:

In an abstract we expect to be told at what timepoints the outcomes were assessed, as well as the duration of the trial. Please add this information to the abstract.

Strengths and limitations of this study

Since randomisation results were concealed until after the consent from the participants were obtained, why was not baseline assessments also performed before revealing randomisation results? This would have reduced the risk of observer bias for the baseline assessments. Observer bias is not possible to be avoided totally in follow-up assessments, but could be avoided for baseline measurements if baseline assessments are perfomed before allocation results are known, se ref.: Catalogue of bias: observer bias; Mahtani, K.,

Spencer, E.A., Brassey, J. & Heneghan, C.;BMJ Evid Based Med,2018. This is one main limitation of the pilot trial, and should be mentioned as such.

Introduction

The introduction is very short, and there is no mentioning of what kind of theoretical assumptions (with references) that undepin the intervention with MAINTAIN. This should included in the introduction.

MAINTAIN seems to be a complex intervention, and it would be interesting to be presented justifications for such a complex intervention, See A new framework for developing and evaluating complex interventions: update of Medical Research Council guidance. Skivington, K., Matthews, L., et al. Bmj 2021

Even if these topics have been elaborated on in a protocol paper, it should not be necessary to read a protocol paper to get an overview of these topics.

Also, did the authors search for systematic reviews on this spesific topic maintaining independence and functions after a fall? This should be stated clearly in the Introduction even if such systematic reviews is not to be found.

Methods

Outcomes

Page 8 Line 31-43. Objectives and outcomes in a pilot RCT are not the same concepts. The authors should be more precise here about what outcome measures they used to achieve the objectives of the PILOT trial. In the abstract this is more precisely formulated, and this should be elaborated on in more detail in the outcome section of the Method section. It should not be necessary to read the attached Statistical Analysis Plan to understand what kind of assessment instruments or scales were performed in the trial.

Discussion:

Page 6 Line 52: I miss the rational for choosing a cluster randomised trial for this intervention. The intervention is rather complex and with up to 19 sessions, so the is very little risk of "contamination" of the intervention toward the patents randomised to a control group in the study site.

Since MAINTAIN is a complex intervention, it is crucial to define clearly its core components and also explain the degree of flexibilty allowed for the implementation of MAINTAIN for the participants and the sites. In general, I miss an in-depth discussion on how to which degree the MAINTAIN intervention is a pragmatic trial/intervention or more explanatory (less pragmatic). Meaning; is it possible to applicate the results from the pilot trial, but also from a future definitive RCT , to real world settings outside the controlled framework of an RCT? This is crucial when you want to take a decision on whether to proceed to a defintive RCT. The MAINTAIN intervention may have several benifits, but is it possible to translate and intergrate this intervention to care in pratice? It might be so, but these questions should be

critically discussed in the Discussion section. See reference: The PRECIS-2 tool: designing trials that are fit for purpose Loudon, K., Treweek, S., Sullivan, F., Donnan, P., Thorpe, K.E. & Zwarenstein, M. BMJ : British Medical Journal 2015 DOI: 10.1136/bmj.h2147

VERSION 1 - AUTHOR RESPONSE

**Reviewer 1**	
Page 7, line 47. Explain what 'PPIE panel' refers to. If this is an expert advisory panel, provide brief details in who was included in the panel and how they were recruited.	Thank you for taking the time to review our manuscript and provide feedback; we greatly appreciate it. The panel involved was a patient and public involvement and engagement (PPIE) panel rather than an expert panel. Members were recruited through Innovations in Dementia. We have now added further detail regarding their role and recruitment on pages 4–5.
Page 7, lines 25-26. Provide further details on who the health professionals were who identified, recruited and consented study participants. Were these members of the research team, employed research assistants, or health professionals working in each of the services the potential participants attended? If participant recruitment and consent was undertaken by health professionals providing services to these clients would pose risk of recruitment bias as well as potential coercion, how was this managed?	We have clarified in the Methods (page 4) that recruitment and consent were undertaken by research staff (e.g., research nurses, assistant psychologists) who were independent of participants' clinical care and not involved in delivering the intervention. Additional detail is also provided in Supplementary File 5.
Page 13, line 11. Insert an additional bracket at the end of (CI:68% to 94&).	Thank you for spotting this typo. We have added the additional bracket.
**Reviewer 2**	
Firstly, Abbreviations must be written out in full the first time they are mentioned. Please check the manuscript carefully for such abbreviations. Since there are some abbreviations not written out in full text.	Thank you for reviewing our manuscript and providing valuable feedback. We have reviewed the manuscript and ensured that all abbreviations are written out in full at first mention.
Title: The title should also reflect design of a study, e.g., Maintaining independence after a fall: a pilot cluster randomised trial of the MAINTAIN intervention for people with dementia	The title has been revised in line with the reviewer and editor feedback to: 'Feasibility of the MAINTAIN intervention to support independence after a fall for people with dementia: a pilot cluster randomised controlled trial in participants' own homes.'
The abstract: In an abstract we expect to be told at what timepoints the outcomes were assessed, as well as the duration of the trial. Please add this information to the abstract.	We have revised the abstract to include the timing of outcome assessments (baseline, 12 weeks and 28 weeks), the duration of the intervention and follow-up (28-week trial period), and the length of the recruitment phase (eight months, September 2023–April 2024). These details are now stated in the abstract.

Strengths and limitations of this study: Since randomisation results were concealed until after the consent from the participants were obtained, why was not baseline assessments also performed before revealing randomisation results? This would have reduced the risk of observer bias for the baseline assessments. Observer bias is not possible to be avoided totally in follow-up assessments, but could be avoided for baseline measurements if baseline assessments are perfomed before allocation results are known, se ref.: Catalogue of bias: observer bias; Mahtani, K., Spencer, E.A., Brassey, J. & Heneghan, C.;BMJ Evid Based Med,2018. This is one main limitation of the pilot trial, and should be mentioned as such.	In this cluster RCT, allocation occurred at the site level and determined whether services were prepared to deliver the intervention. For this reason, research staff necessarily knew their site's allocation before recruitment and baseline assessments. Although allocation was concealed from participants until after consent, baseline assessor blinding was not feasible within this design. We have now clarified this in the manuscript (page 5) and noted the resulting risk of observer bias as a limitation (page 17).
Introduction: The introduction is very short, and there is no mentioning of what kind of theoretical assumptions (with references) that undepin the intervention with MAINTAIN. This should included in the introduction.	We have added a concise summary of the principles underpinning MAINTAIN to the introduction and signposted readers to our process evaluation paper, which provides a fuller account of the programme theory. The introduction remains concise to ensure accessibility for BMJ Open's broad clinical, lay, and research readership, and because the present manuscript focuses on trial outcomes rather than detailed programme theory which is covered in the following paper: https://doi.org/10.1093/ageing/afaf245
MAINTAIN seems to be a complex intervention, and it would be interesting to be presented justifications for such a complex intervention, See A new framework for developing and evaluating complex interventions: update of Medical Research Council guidance. Skivington, K., Matthews, L., et al. Bmj 2021. Even if these topics have been elaborated on in a protocol paper, it should not be necessary to read a protocol paper to get an overview of these topics.	We have added a brief justification for MAINTAIN as a complex intervention, drawing on the updated MRC guidance (Skivington et al., 2021) to explain the need for multiple interacting components (page 4). This summary has been kept concise for accessibility, with readers directed to the protocol and process evaluation papers for fuller detail on intervention development, as it is not feasible to cover all aspects within this manuscript while maintaining focus.
Also, did the authors search for systematic reviews on this spesific topic maintaining independence and functions after a fall? This should be stated clearly in the Introduction even if such systematic reviews is not to be found.	The identification of existing evidence, including systematic reviews relevant to maintaining independence after a fall in people with dementia, was undertaken as part of the intervention development work reported in our process evaluation paper. As noted on page 4, we direct readers to that paper for details about the development of the intervention/programme theory.
Page 8 Line 31-43. Objectives and outcomes in a pilot RCT are not the same concepts. The authors should be more precise here about what	We have revised the Methods section to distinguish more clearly between the

outcome measures they used to achieve the objectives of the PILOT trial. In the abstract this is more precisely formulated, and this should be elaborated on in more detail in the outcome section of the Method section. It should not be necessary to read the attached Statistical Analysis Plan to understand what kind of assessment instruments or scales were performed in the trial.	objectives of the pilot trial and the outcome measures collected to inform feasibility.
Page 6 Line 52: I miss the rational for choosing a cluster randomised trial for this intervention. The intervention is rather complex and with up to 19 sessions, so the is very little risk of "contamination" of the intervention toward the patents randomised to a control group in the study site.	The decision to use a cluster randomised design was pragmatic and intended to minimise contamination. Therapists involved in delivering MAINTAIN may have held routine clinical roles within their services, and there was a risk that skills, strategies, or confidence gained through delivering the intervention could influence the care provided to control participants. Cluster randomisation by site, therefore, ensured clear separation between intervention and usual-care pathways, avoided cross-over of therapeutic approaches within teams, and allowed services to organise delivery without risk of inadvertently exposing control participants to MAINTAIN components.<br><br>We have added a brief justification on page 5.
Since MAINTAIN is a complex intervention, it is crucial to define clearly its core components and also explain the degree of flexibilty allowed for the implementation of MAINTAIN for the participants and the sites. In general, I miss an in-depth discussion on how to which degree the MAINTAIN intervention is a pragmatic trial/intervention or more explanatory (less pragmatic). Meaning; is it possible to applicate the results from the pilot trial, but also from a future definitive RCT , to real world settings outside the controlled framework of an RCT? This is crucial when you want to take a decision on whether to proceed to a defintive RCT. The MAINTAIN intervention may have several benifits, but is it possible to translate and intergrate this intervention to care in pratice? It might be so, but these questions should be critically discussed in the Discussion section. See reference: The PRECIS-2 tool: designing trials that are fit for purpose Loudon, K., Treweek, S., Sullivan, F., Donnan, P., Thorpe, K.E. & Zwarenstein, M. BMJ : British Medical Journal 2015 DOI: 10.1136/bmj.h2147	We have added a concise summary of the principles underpinning MAINTAIN to the Introduction and signposted readers to our process evaluation paper, which provides a fuller account of the intervention's development and programme theory. The present manuscript focuses on trial outcomes rather than detailed theoretical or developmental material.<br><br>With regard to flexibility within the intervention and its pragmatic versus explanatory features, we agree that these are important considerations for understanding the applicability of MAINTAIN in real-world settings. A full assessment of implementation and wider generalisability was beyond the scope of this pilot study; however, our accompanying process evaluation paper addresses some of these issues and will inform decisions about progression to a full trial. A definitive RCT would also provide a greater opportunity to examine the extent to

	which MAINTAIN can be delivered and integrated into routine practice.

VERSION 2 - REVIEW

**Reviewer**	**1**
**Name**	**Chenoweth, Lynn L**
**Affiliation**	**University of New South Wales, School of Psychiatry**
**Date**	**08-Dec-2025**
**COI**	

Thank you for attending to identified issues. The manuscript reads clearly and provides guidance on the future clinical trial of the study intervention and methods.

Tere are just two further issues to be addressed:

1. Table 4. 2nd row. Insert 'completed' in that statement, i.e. 'The person with dementia and carer 'completed' the form'.

2. While the study objective relating to the economic analysis was to develop a cost-effectiveness framework, referenced to the EQ-5D-5L questionnaire and total service costs (intervention and healthcare use), there are no results reported on the actual costs and benefits per participant and in total. Unless there is a brief statement referring to the cost-benefit ratio, or the cost-effectiveness ratio, it is difficult to decipher whether the framework proposed is feasible and/or useful. Would it be possible to provide the results of the economic analysis undertaken?

VERSION 2 - AUTHOR RESPONSE

Reviewer comment	Author comment
1. Table 4. 2nd row. Insert 'completed' in that statement, i.e. 'The person with dementia and carer 'completed' the form'.	Thank you for picking up on this typo, we have now amended it.
2. While the study objective relating to the economic analysis was to develop a cost-effectiveness framework, referenced to the EQ-5D-5L questionnaire and total service costs (intervention and healthcare use), there are no results reported on the actual costs and benefits per participant and in total. Unless there is a brief statement referring to the cost-benefit ratio, or the cost-effectiveness ratio, it is difficult	Thank you for your comment. The purpose of the economic analysis within the study was to build a framework of/for data collection, to assess the feasibility of collecting the data, both in respect of the appropriateness of items included in the data collection and ease of completion of the participant questionnaires, assessed by way of missing data. These results have been reported which

to decipher whether the framework proposed is feasible and/or useful. Would it be possible to provide the results of the economic analysis undertaken?	show good rates of completion of the questionnaires and a small number of changes items which will be changed in a full RCT.<br><br>No analysis of the value of the costs or the cost effectiveness was undertaken given the small number of participants. Whilst the analyses is useful to determine content and structure of the data collection, the values attached to the costs will reveal little given the typical skewed nature of cost data, and may be misleading. As such the value of health and social care resources is not reported.<br><br>We have added a brief section to the discussion:<br><br>‘The embedded framework for economic data collection was successfully implemented and assessed to be both feasible and useful. Whilst the data collected in the study does not inform cost or cost effectiveness, the updated forms will be used in a future full RCT of the intervention for cost and cost effectiveness analysis’.
